# Circulating short and medium chain fatty acids are associated with normoalbuminuria in type 1 diabetes of long duration

**DOI:** 10.1038/s41598-021-87585-1

**Published:** 2021-04-21

**Authors:** Salina Moon, John J. Tsay, Heather Lampert, Zaipul I. Md Dom, Aleksandar D. Kostic, Adam Smiles, Monika A. Niewczas

**Affiliations:** 1 Research Division, Joslin Diabetes Center, One Joslin Place, Boston, MA 02215 USA; 2 Department of Medicine, Harvard Medical School, Boston, MA USA; 3 Department of Microbiology, Harvard Medical School, Boston, MA USA; 4 Present Address: Medicine, Veterans Affairs Boston Healthcare System, Boston, MA USA; 5 Present Address: Department of Family Medicine, Brown University, Providence, RI USA

**Keywords:** Fatty acids, Metabolomics, Biomarkers, Diabetes complications, Type 1 diabetes, Diabetic nephropathy

## Abstract

A substantial number of subjects with Type 1 Diabetes (T1D) of long duration never develop albuminuria or renal function impairment, yet the underlying protective mechanisms remain unknown. Therefore, our study included 308 Joslin Kidney Study subjects who had T1D of long duration (median: 24 years), maintained normal renal function and had either normoalbuminuria or a broad range of albuminuria within the 2 years preceding the metabolomic determinations. Serum samples were subjected to global metabolomic profiling. 352 metabolites were detected in at least 80% of the study population. In the logistic analyses adjusted for multiple testing (Bonferroni corrected α = 0.000028), we identified 38 metabolites associated with persistent normoalbuminuria independently from clinical covariates. Protective metabolites were enriched in Medium Chain Fatty Acids (MCFAs) and in Short Chain Fatty Acids (SCFAs) and particularly involved odd-numbered and dicarboxylate Fatty Acids. One quartile change of nonanoate, the top protective MCFA, was associated with high odds of having persistent normoalbuminuria (OR (95% CI) 0.14 (0.09, 0.23); p < 10^–12^). Multivariable Random Forest analysis concordantly indicated to MCFAs as effective classifiers. Associations of the relevant Fatty Acids with albuminuria seemed to parallel associations with tubular biomarkers. Our findings suggest that MCFAs and SCFAs contribute to the metabolic processes underlying protection against albuminuria development in T1D that are independent from mechanisms associated with changes in renal function.

## Introduction

Diabetic kidney disease (DKD) is characterized by an increase in urinary albumin excretion (albuminuria), which increases risk of renal and cardiovascular complications. Despite broad implementation of renoprotective treatment, the risks of diabetes complications remain high. Therefore, we are faced with a crucial need to elucidate new mechanisms underlying the disease process. Intriguingly, a substantial proportion of subjects with T1D do not develop albuminuria despite having a long duration of diabetes^1–4^. Furthermore, incidence rates of albuminuria in subjects with T1D for 20 years or more eventually start to decline^5–7^. Albuminuria is an intermediate phenotype of DKD preceding kidney failure and increasing cardiovascular mortality. Subjects who, despite a cumulative exposure to T1D of over two decades, did not develop albuminuria or renal function impairment may be considered protected to some extent from progressive DKD.

The majority of etiological studies in diabetic kidney disease have investigated risk factors as determinants of disease progression. Therefore, we can plausibly speculate that studies focusing on protection against diabetic kidney disease development will not only complement the current understanding of risk determinants, but will offer new and invaluable insights into the disease mechanisms.

Over the last decade, metabolomics has emerged from a quiet biochemistry field into mainstream research. Given the metabolic nature of diabetes, metabolomics studies will likely offer insights into the mechanisms underlying chronic diabetic complications^8,9^. Metabolomics is a well-suited tool for such discoveries, as our studies^10,11^ and studies by others^12–16^ have demonstrated. However, the vast majority of these metabolomics studies focused on risk factors of diabetic kidney disease progression.

Subjects with kidney failure possess distinct metabolite profiles compared to subjects with normal renal function. These differences are attributed to the retention of uremic solutes, which have been extensively studied^17,18^. Studies in subjects without renal function impairment are particularly important as they reduce the potential confounding of metabolic changes due to renal accumulation to a minimum.

Albuminuria development has been attributed to different contributions of glomerular, tubular or inflammatory components as reflected by urinary protein biomarker studies^19–22^. To the best of our knowledge, no investigation has yet examined the relationships between albuminuria, urinary biomarkers of diabetic kidney injury and metabolomic aberrations.

The goal of this translational project was to gain a better understanding of the pathophysiological mechanisms underlying protection against progressive diabetic kidney disease in T1D. Our global metabolomics study was particularly designed to identify protective rather than risk patterns as it included subjects with T1D of long duration, normal renal function, and either recent, persistent normoalbuminuria or a broad range of recent, persistent albuminuria. Metabolomic patterns were analyzed in the context of albuminuria and other clinical phenotypes and in the context of urinary protein biomarkers.

## Results

### Clinical characteristics

Our study population comprised 308 subjects with T1D, who were participants of the Joslin Kidney Study (JKS) who maintained normal renal function (mean estimated glomerular filtration rate (eGFR): 101 ± 20 mL/min/1.73 m^2^) over a long duration of diabetes (mean 24 ± 9 years) and had experienced either persistent normoalbuminuria or a wide range of persistent albuminuria within the 2 recent years preceding the metabolomics determinations (Table 1). There were 99 subjects who had normoalbuminuria (median (25th, 75th percentile) albumin/creatinine ratio (ACR): 7 (5, 10) mg/g) and 209 subjects who had albuminuria (median ACR: 665 (393, 1250) mg/g). These two groups had comparable age, duration of diabetes, body mass index (BMI), and statin therapy use. Subjects with normoalbuminuria were more likely to be female, had higher eGFR, more optimal glycemic control, lower blood pressure and cholesterol levels, and were less frequently treated with renin-angiotensin system (RAS) inhibitors than subjects with albuminuria. 92% (n = 91) of subjects with normoalbuminuria and 61% (n = 127) of subjects with albuminuria had eGFR ≥ 90 mL/min/1.73 m^2^.

**Table 1:** Clinical characteristics of the study population.

	Normoalbuminuria (n = 99)	Albuminuria (n = 209)
Age, years	39 ± 11	37 ± 8
Men, n (%)	41 (41%)	120 (57%)
Body mass index, kg/m^2^	25 ± 5	26 ± 5
Systolic blood pressure, mmHg	118 ± 12	129 ± 17
Diastolic blood pressure, mmHg	71 ± 7	78 ± 9
Type 1 Diabetes	100%	100%
Diabetes duration, years	24 ± 9	23 ± 9
HbA_1c_, %	8.8 ± 1.4	9.3 ± 1.7
ACR, mg/g creatinine	7 (5–10)	665 (393–1250)
eGFR, mL/min/1.73 m^2^	110 ± 14	97 ± 21
eGFR category: G1 (≥ 90 mL/min/1.73 m^2^), n (%)	91 (92%)	127 (61%)
eGFR category: G2 (60–< 90 mL/min/1.73 m^2^), n (%)	8 (8%)	82 (39%)
Cholesterol, mg/dL	180 ± 32	215 ± 48
HDL, mg/dL	57 ± 15	56 ± 17
ACE inhibitor/ARB use	19%	67%
Other antihypertensive treatment	8%	16%
Statin use	21%	19%

Study subjects had long duration of Type 1 Diabetes, normal renal function, and persistent normoalbuminuria or a broad range of albuminuria.

*ACR* albumin to creatinine ratio, *HbA*_*1c*_ hemoglobin A_1c_, *eGFR* estimated glomerular filtration rate, *HDL* high density lipoprotein, *ACE* angiotensin-converting enzyme, *ARB* angiotensin II receptor blocker, *n* sample size. Continuous traits are presented as mean (± SD) or median (25th–75th percentile), and binary traits are presented as %. Classification of eGFR followed the guidelines set by NKF/KDIGO^57^.

### Global metabolomic profiling in the multivariable analyses

Serum samples were subjected to mass spectrometry-based metabolomic profiling (Metabolon, Durham, NC). 580 metabolites were detected in at least one study subject. We excluded 228 metabolites that were drug derivatives or infrequently detected. Following analyses were performed on the remaining 352 metabolites that were detected in at least 80% of our study population, which we considered well detectable.

**Figure 1:**
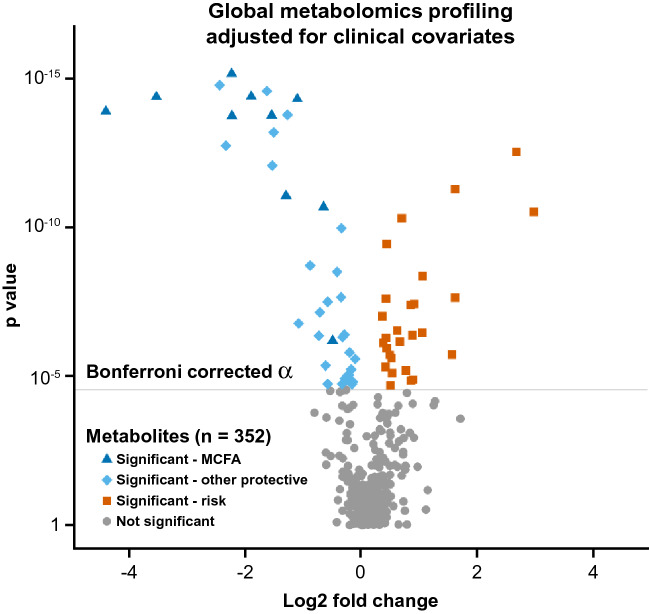
Global analysis of metabolite associations with recent, persistent normoalbuminuria in subjects with long duration of T1D and normal renal function. The analysis is adjusted for clinical covariates. Well-detectable metabolites are shown. The magnitude of the effect is shown as fold change (x-axis) cross-referenced against the strength of the associations represented as significance from the logistic regression model adjusted for clinical covariates (y-axis). The grey line marks the threshold of significance at Bonferroni corrected α = 0.000028. Each point represents an individual metabolite. Dark blue triangles mark significant Medium Chain Fatty Acids. Light blue diamonds mark other significant protective metabolites. Orange squares mark significant risk metabolites. *MCFA* Medium Chain Fatty Acid.

We examined associations between 352 well-detectable metabolites and albuminuria status in logistic regression analyses after adjustment for multiple testing. In the crude model, 94 metabolites were significantly associated with albuminuria [Bonferroni corrected α = 0.000028 (p = 0.01/352)]. Of these, 65 metabolites remained significant in the multivariable analysis (after further adjustment for clinical covariates: age, gender, hemoglobin A_1c_ (HbA_1c_), and eGFR) (Fig. 1).

This study was designed to detect protective factors in particular. Indeed, in our global profiling analysis, 38 of these metabolites displayed a pattern associated with a recent, 2-year history of protection from albuminuria, characterized by their higher concentrations in subjects with recent, persistent normoalbuminuria than in subjects with recent, persistent increased albumin excretion in subjects with T1D of long duration and preserved renal function. By contrast, we identified only 27 metabolites constituting a risk pattern. Among 38 protective metabolites, Medium Chain Fatty Acids (MCFAs) accounted for the most abundant biochemical subclass (26%), marked by dark blue triangles in Fig. 1. The next most abundant biochemical subclass of the protective factors consisted of four aromatic amino acids.

**Figure 2:**
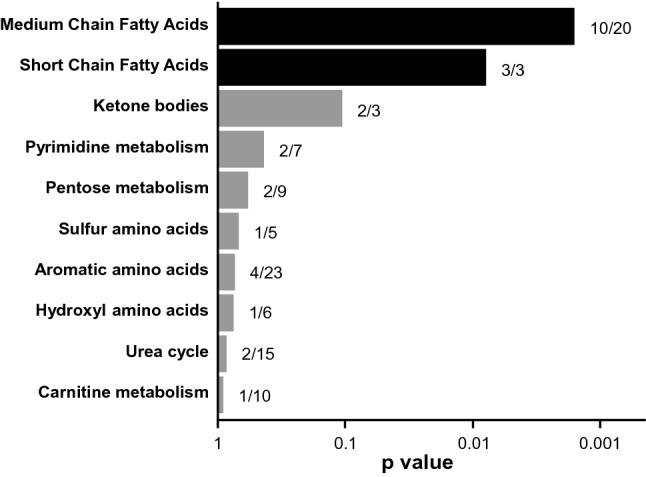
Enrichment of protective metabolites in select biochemical subclasses. The fraction of metabolites in each subclass that are protective is presented to the right of each bar. One-tailed Fisher’s exact test p values are shown following their logarithmic base 10 transformation (x-axis). Blue bars mark metabolite subclasses that are significantly enriched. The top ten subclasses are shown.

The protective metabolites were significantly enriched in MCFAs (Fisher’s exact test p = 0.0016). Half of all MCFAs were associated with protection against albuminuria, contributing to 26% (n = 10) of the 38 protective metabolites. Although there were only three Short Chain Fatty Acids (SCFAs), they were all associated with our outcome of interest (Fisher’s exact test p = 0.0079). No other metabolite classes were significantly enriched (Fig. 2).

Logistic regression analyses are highly appropriate models to examine independence of the metabolite from the clinical covariates. Nevertheless, they have limited capacity to examine multiple metabolites in the same model. Therefore, our next step was to turn to the Random Forest analysis, an ensemble method that is well suited to examine multiple covariates (metabolites) simultaneously in the context of a binary outcome and handles well-correlated data. Our Random Forest classification model was built upon the 352 well-detectable metabolites. The contribution of a metabolite to the model was evaluated with the mean GINI index decrease. Remarkably, of the top 30 classifiers shown in Supplementary Fig. S1, all but two were significantly associated with albuminuria in the multivariable logistic analyses. The top 20 contributing metabolites comprised 15 protective factors and 5 risk factors previously identified in the multivariable logistic analyses. Furthermore, the top 11 SCFAs and MCFAs classifiers in the Random Forest analysis were concordant with the most robustly associated metabolites of these subclasses in the logistic regression analyses. Among the other protective metabolites in the top 20 classifiers, two were Long Chain Fatty Acids (LCFAs) of relatively short (13 and 14 carbon chain) lengths.

### Short and Medium Chain Fatty Acids and persistent normoalbuminuria phenotype

**Figure 3:**
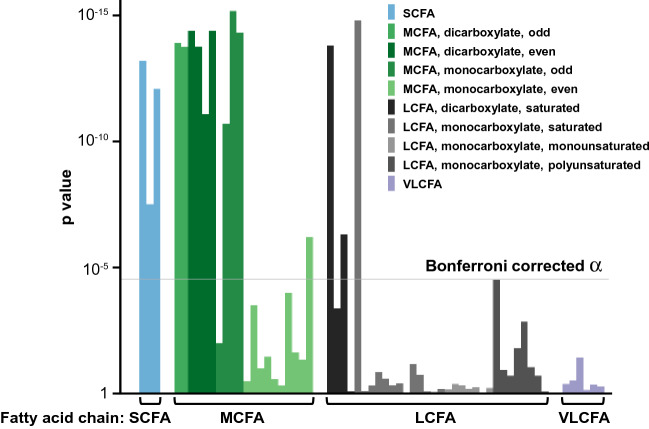
Comprehensive analysis of Fatty Acid associations with a recent history of persistent normoalbuminuria phenotype. The needle plot depicts p values obtained with the logistic regression analysis adjusted for clinical covariates following their logarithmic base 10 transformation. Each needle represents an individual Fatty Acid. Fatty Acids are ordered by chain length categories, followed by structural features. The grey line marks the threshold of significance at Bonferroni corrected α = 0.000028. *SCFA* Short Chain Fatty Acid, *MCFA* Medium Chain Fatty Acid, *LCFA* Long Chain Fatty Acid, *VLCFA* Very Long Chain Fatty Acid.

Medium, Short, and select Long Chain Fatty Acids were metabolites that were robustly associated with protection from persistent albuminuria in the logistic analyses (MCFAs), demonstrated biochemical subclass enrichment (SCFAs and MCFAs), and/or belonged to the top Fatty Acids in the Random Forest analysis (MCFAs and select LCFAs). Therefore, we decided to examine all Fatty Acid metabolites in greater detail. Our metabolomic array included 69 Fatty Acids, 61 of which were well detectable. In the multivariable logistic analyses adjusted for clinical covariates and for multiple testing, 100% of SCFAs, 50% of MCFAs, 9% of LCFAs, and 0% of Very Long Chain Fatty Acids (VLCFAs) were significantly associated with protection from albuminuria (Fig. 3, Supplementary Table S1).

**Figure 4:**
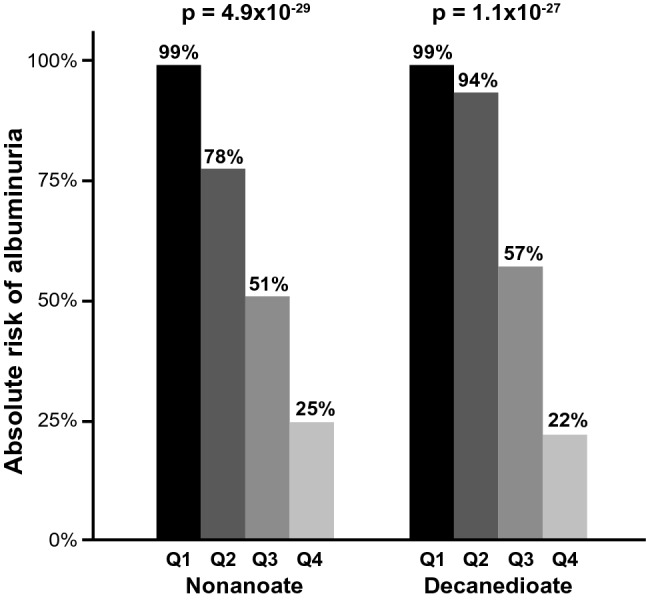
Absolute risks of albuminuria by quartiles (Q1–Q4) of top MCFAs: nonanoate (left) and decanedioate (right). Chi-square test p values are shown.

Odds of protection with a recent, 2-year history of persistent normoalbuminuria were robustly significant for 13 Short and Medium Chain Fatty Acids with p values ranging from 6.3 × 10^–7^ to 7.0 × 10^–16^ (Table 2). Odds of protection were almost 4 times higher per one quartile increase in the circulating levels of the top SCFA, 2-hydroxyglutarate (OR (95% CI) 0.25 (0.18, 0.36), p < 10^–13^). The absolute risk of a recent, 2-year history of albuminuria was 99% in subjects with low levels of the top MCFA, nonanoate (quartile 1), and risk decreased to 25% in subjects with high levels of nonanoate (quartile 4). Patterns were highly similar for the second top MCFA, decanedioate (Fig. 4).

**Table 2:** Short and Medium Chain Fatty Acids associations with recent, persistent normoalbuminuria in subjects with a long duration of T1D and normal renal function.

Metabolite	Chemical formula	Model 1			Model 2	
		OR (95% CI)	Nominal p	Bonferroni p	OR (95% CI)	Nominal p
**Short Chain Fatty Acid (SCFA)**						
Glutarate	C_5_H_8_O_4_	0.28 (0.20, 0.40)	8.4E−13	3.0E−10	0.33 (0.21, 0.52)	1.8E−6
2-hydroxyglutarate	C_5_H_8_O_5_	0.25 (0.18, 0.36)	6.5E−14	2.3E−11	0.29 (0.18, 0.46)	1.8E−7
4-hydroxycarboxylate	C_4_H_8_O_3_	0.45 (0.34, 0.60)	3.1E−8	1.1E−5	0.50 (0.34, 0.73)	3.3E−4
**Medium Chain Fatty Acid (MCFA)**						
**Dicarboxylate**						
Odd-numbered						
Nonanedioate	C_9_H_16_O_4_	0.21 (0.14, 0.31)	1.3E−14	4.5E−12	0.23 (0.13, 0.39)	6.4E−8
Undecanedioate	C_11_H_20_O_4_	0.19 (0.13, 0.30)	1.9E−14	6.5E−12	0.18 (0.10, 0.33)	1.5E−8
Even-numbered						
Hexanedioate	C_6_H_10_O_4_	0.33 (0.24, 0.46)	8.8E−12	3.1E−9	0.36 (0.23, 0.57)	1.2E−5
Octanedioate	C_8_H_14_O_4_	0.18 (0.12, 0.28)	4.1E−15	1.5E−12	0.18 (0.10, 0.32)	9.0E−9
Decanedioate	C_10_H_18_O_4_	0.16 (0.10, 0.25)	4.2E−15	1.5E−12	0.15 (0.08, 0.29)	1.1E−8
Dodecanedioate	C_12_H_22_O_4_	0.21 (0.14, 0.32)	1.8E−14	6.2E−12	0.23 (0.14, 0.39)	3.8E−8
**Monocarboxylate**						
Odd-numbered						
Heptanoate	C_7_H_14_O_2_	0.32 (0.23, 0.44)	2.1E−11	7.3E−9	0.30 (0.19, 0.47)	1.4E−7
Nonanoate	C_9_H_18_O_2_	0.14 (0.09, 0.23)	7.0E−16	2.5E−13	0.14 (0.07, 0.27)	2.7E−9
Undecanoate	C_11_H_22_O_2_	0.20 (0.13, 0.30)	5.0E−15	1.7E−12	0.23 (0.13, 0.39)	6.6E−8
Even-numbered						
Octanoate	C_8_H_16_O_2_	0.48 (0.37, 0.64)	6.3E−7	2.2E−4	0.56 (0.38, 0.82)	3.0E−3

Logistic regression analyses are adjusted for clinical covariates. Model 1 is corrected for age, gender, HbA_1c_, and eGFR. Model 2 is additionally corrected for systolic blood pressure, diastolic blood pressure, cholesterol, HDL, ACE inhibitor/ARB use, other antihypertensive treatment, and statin use. Effect sizes are shown per one quartile change. Protective Short and Medium Chain Fatty Acids are ordered by structural features and subsequently by the number of carbons. *OR* odds ratio, *CI* confidence intervals. For more comprehensive logistic analyses of all Fatty Acids, please refer to Supplementary Table S1.

We have further compared the contributions of dicarboxylate and monocarboxylate metabolites among significant Fatty Acids. Although only 28% of MCFAs were dicarboxylate, they accounted for 60% of the protective metabolites of this subclass. Similarly, dicarboxylates comprised only 12% of all LCFAs but strikingly accounted for 67% of protective metabolites of the respective subclass.

We analyzed the parity of carbon numbers as well. Out of 4 protective monocarboxylate MCFAs, 75% were odd-numbered, even though only 22% of all monocarboxylate MCFAs were odd-numbered. The differences were less striking for LCFAs. One out of 3 protective LCFAs was odd-numbered, even though only 19% of all LCFAs were odd-numbered. Parity was not a distinguishing factor among Short and dicarboxylate Medium Chain Fatty Acids as they were all protective.

Analysis of the related beta estimates from the crude and multivariable models revealed that neither eGFR nor HbA_1c_ confounded associations (Δβ < 20% for all comparisons). Further adjustment by an extended set of clinical covariates is presented in Table 2.

Subsequently, we examined the orthogonal relationships between protective Fatty Acids and other protective metabolites in the cluster analysis shown in Supplementary Fig. S2. We identified two major clusters: Cluster 1 comprised all Fatty Acids, whereas Cluster 2 grouped all but one non-lipid metabolites. The patterns were most tightly grouped for MCFAs, as reflected by the short distances. The subject-level clustering revealed that 63% of subjects in Cluster 1 were with persistent normoalbuminuria, as opposed to 14% and 4% in Clusters 2 and 3, respectively (Supplementary Fig. S2).

We evaluated the relationships between all well-detectable Fatty Acids (n = 61) and urinary biomarkers of kidney injury and glycemic control by examining Spearman correlation coefficients (Supplementary Fig. S3). Interestingly, correlations with Fatty Acids displayed similar patterns with ACR to those with plasma and urinary KIM-1 (Kidney Injury Molecule 1). By contrast, Fatty Acids showed weaker correlations with IgG2 (Immunoglobulin G2). The associations were also weak for such clinical phenotypes as eGFR and HbA_1c_.

The associations from the crude and multivariable models of protective and risk metabolites with our outcome of interest are shown in Supplementary Table S2. The 27 risk metabolites were measured at higher concentrations in subjects with albuminuria in comparison with subjects with normoalbuminuria. The most abundant biochemical subclasses comprised lysolipids, oxidized cholesterols, peptides, and eicosanoids.

Among recently published metabolomics studies, we have identified one particular study that had a lot of similarities in regards to the clinical phenotypes and used the same metabolomics platform as our study. The report by Haukka et al. was based on a medium-sized case–control study nested within the prospective Finnish Diabetic Nephropathy Study (FinnDiane) cohort with T1D^23^. Subjects with T1D, normoalbuminuria and normal renal function at baseline were followed for a median of 7 years to identify incident microalbuminuria cases. The clinical phenotype of this study population resembled to a large extent our normoalbuminuria group, particularly in terms of diabetes duration, renal function and ACR levels. Therefore we have comprehensively compared patterns of metabolite associations with albuminuria phenotypes between Haukka et al.^23^ and our study. There were 42 metabolites mutually reported in both studies. The pattern concordance between the two studies was evaluated at α = 0.05. Almost half of these metabolites (45%, n = 19) revealed a concordant pattern between the longitudinal FinnDiane study and our cross-sectional Joslin study. Uremic solutes, lysolipids, and certain other metabolites were among highly concordant metabolites. Concordant patterns were also noted for branched chain fatty acids but not in two straight chain fatty acids (glutarate and octanedioate). In Supplementary Table S3, we provide the patterns of the associations for the 42 metabolites in both studies together with effect estimates in our study.

## Discussion

Our study was conducted in a study population of 308 subjects with T1D of long duration, normal renal function, and recent, persistent normoalbuminuria or a wide range of recent, persistent albuminuria. In the current study, global metabolomic profiling identified 38 metabolites associated with protection offered by recent, persistent normoalbuminuria with normal renal function in T1D and 27 risk factors, respectively. Medium Chain Fatty Acids were the most abundant biochemical subclass in the set of protective metabolites, and enrichment in MCFAs was highly significant. Associations were highly robust in the analysis adjusted for clinical covariates and for multiple testing. A parallel machine learning approach, Random Forest, concordantly pointed to MCFAs as top classifiers of the albuminuria phenotype. Overall, the set of protective metabolites included Fatty Acids with 4 to 14 carbons (Short, Medium, and “shorter” Long Chain Fatty Acids). Associations with albuminuria phenotype were particularly robust for Fatty Acids that were dicarboxylate or odd-numbered.

MCFAs are a subclass of Fatty Acids with a chain containing 6 to 12 carbons. MCFAs have been suggested to yield protective properties in a number of diseased conditions: Type 2 Diabetes (T2D)^24^, chronic kidney disease (CKD) and albuminuria^15,25^, cardiovascular disease (CVD)^26,27^, and obesity^28^.

Untargeted metabolomics in a small cross-sectional study of subjects from the Kooperative Gesundheitsforschung in der Region Augsburg Follow-up von S3 (KORA F3) cohort identified a few MCFAs inversely associated with T2D in comparison with healthy individuals^24^. Another untargeted metabolomic study of a small number of subjects with T2D identified a MCFA (nonanoate) as the top protective factor against incident coronary artery disease^26^. A short-term interventional study in just over a dozen T2D subjects on a diet rich in MCFAs demonstrated a significant improvement in myocardial velocity in comparison with baseline and with a diet rich in LCFAs^27^. Another small study found two MCFAs as the top ranking classifiers of subjects without CKD from those with moderate CKD^25^. In a medium-sized prospective study in subjects with T1D from Steno Diabetes Center, one MCFA (octanoate) was, similar to our study, inversely associated with albuminuria phenotype and eGFR decline^15^. However, in a FinnDiane study of subjects with T1D and normoalbuminuria two of our main protective SCFAs (glutarate) and MCFAs (octanedioate) were risk factors of incident microalbuminuria^23^. Two other studies performed in subjects with more advanced kidney disease from the Chronic Renal Insufficiency Cohort (CRIC) evaluated a number of SCFAs and MCFAs for prospective eGFR-based outcomes. No associations were identified, except for heptanedioate being a risk factor of CKD progression in one of the CRIC studies^14,29^. The differences between these latter studies and ours may stem from different study design, dissimilarities in diabetic kidney disease stages or variations in cohorts.

MCFAs have well-documented antimicrobial properties^30^, and research has suggested they may reduce inflammation^31^ and provide more readily available energy in the mitochondria than LCFAs^32^. When it comes to potential sources of MCFAs, studies point to gut microbial species or dairy intake^25,33–36^.

Our study pointed to odd-numbered MCFAs, such as nonanoate and undecanoate, as they exhibited particularly strong protective patterns against albuminuria. Other studies reported that odd-numbered Fatty Acids could be considered protective against T2D risk (European Prospective Investigation into Cancer and Nutrition (EPIC)-Norfolk study) and against CVD (EPIC-InterAct)^33,37^.

By contrast, associations of even-numbered MCFAs with albuminuria phenotype were much weaker than with odd-numbered ones in our study. Our findings remain in concordance with recent advances regarding coconut oil and CVD risk. The most abundant Fatty Acid in coconut oil is dodecanoate, an even-numbered MCFA, and combined with 14 and 16 carbon length LCFAs comprise approximately 70% of this oil content^38^. Some time ago, coconut oil gained popularity for its alleged health benefits that were commonly ascribed to its high MCFA content. Nevertheless, a recent, comprehensive meta-analysis of several clinical trials on the effect of coconut oil has found that its consumption is associated with unfavorable lipid profiles (increased plasma levels of total and LDL cholesterols)^39^. Odd-numbered and even-numbered MCFAs exert distinct downstream effects on cellular energy metabolism. Beta-oxidation of odd-numbered MCFAs generates acetyl-CoA similarly to even-numbered MCFAs, but in addition, they generate anaplerotic propionyl-CoA^40^.

Additionally, our study revealed stronger patterns of protection for dicarboxylate MCFAs in comparison with monocarboxylate MCFAs. Every dicarboxylate MCFA was protective in our study. Furthermore, among the dicarboxylate Fatty Acids of any chain length, 83% were protective, whereas only 13% of monocarboxylate Fatty Acids displayed a protective pattern. A dicarboxylic acid lipid is a metabolite containing two carboxyl groups, in comparison with a monocarboxylic acid that contains only one. Dicarboxylate Fatty Acids have been tested as a treatment for diabetes in small animal and human studies^41^. Two small interventional studies of dicarboxylate MCFAs revealed beneficial glycemic profiles^42,43^. Another interventional study showed that treatment with a glucose-lowering, reno- and cardioprotective sodium-glucose co-transporter 2 inhibitor (SGLT2i), dapagliflozin, reduced plasma octodecanedioate levels^44^.

Short Chain Fatty Acids comprised the second top enriched biochemical subclass inversely associated with previous progression of albuminuria in our study. Although only four SCFAs were measured, they were all associated with historical protection from albuminuria. SCFAs are a subclass of Fatty Acids with five or fewer carbon length chain, and have been implicated in potential beneficial roles in diabetes, renal function, CVD, and obesity^15,45^. A few small and short-term interventional studies demonstrated that either dietary supplementation or colonic infusion with SCFAs resulted in a reduction in weight and blood pressure, improved beta cell function, and lower levels of postprandial insulin^46–51^.

SCFAs are the main products of the fermentation of non-digestible carbohydrates by the gut microbiome^52^. A study found that *Escherichia coli*, a gut bacterial species, contains a hydroxylase that converts glutarate to 2-hydroxyglutarate, two SCFAs identified in our study as protective^53^. A metabolomic study in subjects with T2D has also demonstrated protective associations against albuminuria for 4-hydroxycarboxylate, another SCFAs also identified in our study, although these determinations were performed in urine, as opposed to our metabolomic profiling that was performed in plasma^54^. We must acknowledge that the most commonly evaluated SCFAs in gut microbiome studies, namely acetate, propionate, and butyrate, were not detected on the metabolomic platform used in our study^55^.

Albuminuria and renal function trajectories constitute two major intermediate phenotypes of diabetic kidney disease. The two only partially overlap when it comes to kidney failure risk^2,56,57^. It is plausible to assume that the metabolomic signatures may also only partially overlap between the two. Interestingly, our observed associations between lipid metabolites, specifically Fatty Acids, and albuminuria were not confounded by renal function. Furthermore, the protective Fatty Acids clustered together and separately from non-lipid metabolites. These findings suggest that circulating protective lipid metabolites associated with albuminuria reflect mutual underlying mechanisms that are distinct from the mechanisms reflected by other non-lipid metabolomic disturbances and also from those associated with renal function phenotype.

Metabolomic studies in the diabetic kidney field concentrated on eGFR-based outcomes in diabetic or chronic kidney disease, yet we observed concordant patterns in our study. A number of uremic solutes, including pseudouridine and C-glycosyltryptophan, were robustly associated with our outcome of interest. Similar risk patterns for progressive DKD regarding uremic solutes were also reported in several studies evaluating eGFR-based outcomes^10,11,13,23,58^. In addition, lysolipids, dipeptides and oxidized cholesterol derivatives displayed risk patterns in our study and several others^23,59–61^.

Some efforts have also been undertaken to examine albuminuria-based outcomes^23,62–64^. One prospective case–control study examined progression from normoalbuminuria to microalbuminuria using the same metabolomics platform as ours in a subset of the FinnDiane cohort with a highly comparable clinical phenotype to our study population^23^. There was a large degree of concordance in the metabolites reported in both studies, particularly uremic solutes and lysolipids. Glutamine and 1,5-anhydroglucitol (1,5-AG) were concordant metabolites that displayed a protective pattern reflected in this^23^ and other studies^65–70^. In an independent report by Pena et al., glutamine was inversely correlated with longitudinal changes in ACR, although it was measured in urine of subjects with T2D^67^. Functional studies of glutamine administration in the sepsis-induced or cisplatin-induced kidney injury models also demonstrated renoprotection^65,66^. 1,5-AG was shown to inversely associate with albuminuria in the Atherosclerosis Risk in Communities (ARIC) Study comprising 1600 older subjects^69^. Furthermore, in the ARIC Study of over 10 thousand subjects, low 1,5-AG levels were shown to be associated with incident CKD and end-stage kidney disease, although the latter was not independent of glycemia^68,70^.

Nevertheless, most of these studies have focused on risk factors of disease progression. Our study confirms previously frequently reported risk metabolites of diabetic kidney complications discussed above. Our study expands this body of knowledge further to a comprehensive evaluation of metabolites offering protection against the progressive disease course, in our case against albuminuria development.

To the best of our knowledge, our study is the only one that aimed to examine relationships between the circulating metabolome and biomarkers of diabetic kidney injury. Our findings suggest that protection reflected by SCFAs and MCFAs involve tubular mechanisms as captured by urinary and plasma KIM-1, and to a much lesser degree, by glomerular or inflammatory mechanisms. Functional studies demonstrated a relationship between Fatty Acid handling and tubular injury. Their involvement suggests that impaired fatty acid oxidation and accumulation of longer chain Fatty Acids may lead to tubular injury. A study on tubule epithelial cells has demonstrated that inhibition of fatty acid oxidation induced a fibrotic phenotype^71^. Furthermore, improving fatty acid oxidation in mouse models of fibrosis ameliorated tubulointerstitial fibrosis^71^. Proximal tubular epithelial cells treated with palmitate, a 16 carbon length LCFA, experienced significantly higher rates of apoptosis compared to those treated with control bovine serum albumin^72^. Our earlier biomarker study demonstrated that urinary levels of KIM-1 were associated with albuminuria regression^20^.

Finally, the strengths and limitations of the current study should be considered. Our study population featured strong ascertainment of albuminuria and a relatively large sample size. Importantly, the study subjects had normal renal function, so changes in the metabolite levels due to renal function impairment were unlikely.

Our study design included subjects with T1D of long duration, and it was therefore particularly designed to examine protective factors. Unlike the many studies referenced previously, ours is the first to include persistent normoalbuminuria and a broad range of persistent albuminuria in a large cohort of subjects whose renal function remained normal over a long course of T1D. Although our study was cross-sectional by design, as the biospecimens for metabolomics profiling were sampled at a single timepoint, our study also incorporated certain features of longitudinal design when it comes to the selection of the study group. Our study population, particularly the normoalbuminuria group, was naturally “enriched” in protected subjects, meaning that after almost 25 years of diabetes duration these subjects did not develop more progressive forms of DKD (renal function impairment, kidney failure or fatal events). Indeed, our study identified a higher number of metabolites associated with historical protection from albuminuria in comparison with risk factors, a pattern that is infrequently seen in other study designs. The choice of the comparator group in studies on protection may also be arduous. Our comparator group comprised age, diabetes duration and eGFR-balanced subjects with T1D, albuminuria and normal renal function. Although this group may also be partially protected from progressive disease, importantly, this design minimizes possible confounding by renal function impairment or other key characteristics.

The concept of protection in our study design is further supported by the natural history of DKD course. A study based on Joslin Kidney Study subjects with T1D revealed that the risk of persistent proteinuria peaks during the second decade and declines afterwards^6,7^. Correspondingly, a large prospective Steno cohort study of subjects with T1D reported that the incidence rates of proteinuria are high 10 years into the T1D course, but they are lower in the third decade of T1D duration^5^. A study of subjects with T1D for more than 50 years also demonstrated that a high proportion of this population (70%) remained free of albuminuria^73^. The natural history of DKD data supports the thesis that only a subset of subjects with T1D will develop progressive DKD. This decline in incidence rates over time may result from the fact that complications due to DKD had already occurred in the majority of susceptible subjects earlier in the disease course or that they may be attributed to the protective effects of clinical factors such as optimal glycemic or blood pressure control^5–7,74–76^. We may postulate further that the Short and Medium Chain Fatty Acid alterations observed in our study may constitute additional factors offering protection.

The untargeted metabolomic approach taken in our study utilized state-of-the-art technology allowing for an examination of comprehensive profiles of metabolites. Our study also featured a robust computational approach evaluating an effect independence from the clinical covariates, a rigorous adjustment for multiple testing, and biostatistical methods (multivariable logistic regression) used in parallel with complementary machine learning tools (multivariable Random Forest). Biostatistical models are easily interpretable, and we can evaluate measures of uncertainty and confounding. Nevertheless, they allow for only a limited number of independent variables. Random Forest is an ensemble machine learning method belonging to the decision tree family. It is similarly well suited for binary outcomes like logistic regression. Yet, Random Forest performs better than logistic analysis in the presence of a large number of independent variables and deals well with correlated data, threshold effects or interactions. It also typically offers superior model accuracy. On the other hand, the model itself is less interpretable^77^.

Although the nature of our study does not allow for inferring causality, it does allow for identifying metabolomic mechanisms underlying the protective phenotype from albuminuria. Replication of our findings in an independent dataset is required to validate these associations. Our findings may not be generalizable to clinical phenotypes in subjects with T2D or other non-diabetic kidney diseases. Our metabolomic platform did not have capabilities of measuring complex lipids.

Our global metabolomic profiling study has identified for the first time Medium and Short Chain Fatty Acids as robust metabolites of a protective pattern against albuminuria development in T1D. The associations seemed to be independent from other major intermediate phenotypes of diabetic kidney disease such as renal function or glycemic control. Our biomarker results suggest further that protective mechanisms involve tubular mechanisms. Further functional and dietary interventional studies are warranted to ultimately define the role of MCFAs and SCFAs in the diabetic kidney disease course.

## Materials and methods

### Study population

Our research project followed the principles of the Declaration of Helsinki. The Committee on Human Subjects of Joslin Diabetes Center approved the informed consent, recruitment, and examination procedures for this study. Informed consent was obtained from all study participants. Our cross-sectional study population comprised 308 subjects with T1D, who were participants of the Joslin Kidney Study. Study subjects were recruited from approximately 3,500 adult patients (95% Caucasian) with T1D who received care at the Joslin Clinic (Boston, MA) between 1991 and 2009^78–80^. At enrollment, trained recruiters performed a structured interview and brief physical examination. Blood and urine specimen were collected from 1,884 subjects at that time and stored at -80° C until analysis. Eligibility criteria for our current study included residence in New England, age between 21 and 54 years at enrollment, T1D diagnosis before age 40, and eGFR ≥ 60 mL/min/1.73 m^2^. From this population, we excluded subjects who were on dialysis, received a kidney transplant, had a history of HIV or hepatitis C virus infection, or had other significant comorbidities. Finally, after exclusion of subjects with CKD stages 3–4, 308 subjects had serum samples available for the current study.

### Ascertainment of the albuminuria status

Immunonephelometry was used to measure spot urinary albumin concentration with the BN ProSpec System (Dade Behring, Newark, DE) (intra-assay and inter-assay coefficients of variation (CV): 4% and 6%, respectively). The Jaffe modified picrate method was used to measure urinary creatinine with the Ciba Corning 550 Express Plus Chemistry Analyzer (intra-assay and inter-assay CV: 5% and 3%, respectively). ACR was defined as the concentration of urinary albumin normalized to the concentration of urinary creatinine. In order to account for the high intra-individual variability of ACR over time, ascertainment of recent, 2-year history of persistent normoalbuminuria or albuminuria status was based on the geometric mean of repeated measurements of ACR taken during the two years preceding the metabolomic profiling. Normoalbuminuria was defined as a geometric mean of ACR measurements < 30 mg/g, and albuminuria was defined as a geometric mean of ACR measurements ≥ 30 mg/g.

Albuminuria and eGFR categories were based on the nomenclature from The National Kidney Foundation (NKF) and Kidney Disease: Improving Global Outcomes (KDIGO)^57^.

### Metabolomic profiling

The detailed methods of metabolomic profiling were reported in previous studies^10,11^. Serum samples were subjected to a mass spectrometry-based global metabolomic platform (Metabolon, Durham, NC) that utilized gas and liquid based chromatography in both positive and negative modes^81^. The platform library comprised approximately 2,800 metabolites (Supplementary Table S4).

### Biomarkers

The detailed methods of measuring a urinary biomarker of glomerular damage, IgG2, and plasma and urinary biomarkers of tubular damage, KIM-1, were reported in previous studies^19,22^. Concentrations of the biomarkers were measured on the Luminex platform. Human Immunoglobulin Isotyping Panel (Millipore; Billerica, MA) and Human Magnetic Kidney Biomarker Panel (R&D Systems; Minneapolis, MN) were used to measure IgG2 and KIM-1, respectively. Urinary biomarkers were normalized to urinary creatinine. Measurements of urinary IgG2, urinary KIM-1, and plasma KIM-1 levels were available for 204, 201, and 242 subjects, respectively.

### Data analysis

Clinical variables were compared between the normoalbuminuria and albuminuria groups by analysis of variance or with a chi-square test, as applicable. Measurements of 98% of well-detectable metabolites departed from a normal distribution [Shapiro–Wilk test, p < 0.05, median (25th percentile, 75th percentile) coefficient of skewness: 2.1 (1.3, 3.7) and of kurtosis: 7.0 (2.7, 20.1)], thus a quantile change approach was used in logistic regression analyses. Well-detectable metabolite concentrations were transformed to percentile ranks, and their effects on albuminuria per one quartile change were analyzed by logistic regression in the crude model and the model adjusted for age, sex, HbA_1c_, and eGFR. Protective Short and Medium Chain Fatty Acids were analyzed in the model additionally adjusted for systolic and diastolic blood pressure, cholesterol level, HDL level, and use of ACE-inhibitor, ARB, other antihypertensive medications, and statin. Values of these clinical covariates were missing for less than 15% of the study population. We did not use any imputation for missing values. Adjustment for multiple testing was performed with a threshold of significance at Bonferroni corrected α = 0.000028 (0.01/352). Odds ratios may be converted to β estimates using the formula: β = ln(OR).

Logistic regression of metabolites belonging to the Fatty Acid biochemical subclass that were not well detectable was performed as follows. Values of metabolites that were detected in at least 50% but less than 80% of our study population were separated into two quantiles: those above the median, and those below or equal to the median. Values of metabolites that were detected in less than 50% of the study population were analyzed as categorical variables: those that were detected, and those that were not detected. All other statistical analyses were performed on well-detectable metabolites.

Right-sided Fisher exact test was used to determine enrichment in the biochemical subclasses. The enrichment test was performed for subclasses that had at least 2 metabolites associated with protection from albuminuria. The volcano plot was based on the fold change (ratio) of the mean concentration of each metabolite in subjects with albuminuria over the mean value in subjects with normoalbuminuria. Colors in the volcano plot were chosen from a colorblind-friendly palette^82^. In Fig. 3, the needle plot was based on the p values obtained in the adjusted logistic regression model of Fatty Acids. In Supplementary Fig. S3, the needle plots were based on correlation coefficients obtained in Spearman correlation analyses between Fatty Acids and classical markers of glomerular damage (ACR and IgG2), clinical phenotypes (eGFR and HbA_1c_), and markers of tubular injury (plasma and urinary KIM-1). We evaluated correlations in 155 subjects (40 with normoalbuminuria and 115 with albuminuria) for whom all those measurements were available. The hierarchical clusters and heat map were generated using the Ward method based on metabolite concentrations transformed to percentile ranks. Random Forest models of metabolites to differentiate albuminuria strata were built using Breiman’s classic algorithm with 600 trees, and the number of metabolites randomly sampled as candidates at each split was defined as the square root of the total number of classifiers (n = 352), which in these models was 18. The dataset was split into a training set (70%) and a validation set (30%) in order to perform an internal validation. The contribution of a metabolite as a classifier was determined by the mean decrease in GINI index of the model when the metabolite is removed from the classification set. The absolute risks of albuminuria per one quartile of each of the top two MCFAs, nonanoate and decanedioate, were analyzed by chi-square test.

Statistical analyses were performed with SAS 9.4 (SAS, Cary, NC). Hierarchical cluster analysis was performed with JMP Pro 14.0.0 software (SAS, Cary, NC). Plots were generated with the R package ggplot2, and Random Forest models were built with the R package randomForest, in R, version 3.5.0 (R Core Team, 2018)^83,84^.

## Supplementary Information

Supplementary Information.

## Data Availability

The datasets generated and/or analyzed during the current study are available from the corresponding author upon reasonable request.
