# Supplementary material for: Circulating short and medium chain fatty acids are associated with normoalbuminuria in type 1 diabetes of long duration
Source: Sci Rep. 2021 Apr 21;11:8592. doi: 10.1038/s41598-021-87585-1 (PMC8060327; doi:10.1038/s41598-021-87585-1)
Supplement: Supplementary file 1 — Supplementary Information. [file 41598_2021_87585_MOESM1_ESM.pdf]

Supplementary information

Circulating short and medium chain fatty acids are associated with normoalbuminuria in type 1 diabetes of long duration

Salina Moon[1], John J. Tsay[1,2,3], Heather Lampert[1,2,4], Zaipul I. Md Dom[1,2], Aleksandar D. Kostic[1,2,5], Adam Smiles[1,†], Monika A. Niewczas[1,2] *

[1]Research Division, Joslin Diabetes Center, Boston, MA.
[2]Department of Medicine, Harvard Medical School, Boston, MA, USA.
[3]Current Affiliation, Medicine, Veterans Affairs Boston Healthcare System, Boston, MA.
[4]Current Affiliation, Department of Family Medicine, Brown University, Providence, RI.
[5]Department of Microbiology, Harvard Medical School, Boston, MA, USA.
[†]Deceased

*Corresponding author: monika.niewczas@joslin.harvard.edu

Supplementary Figure S1. Top thirty metabolites as classifiers of recent, persistent normoalbuminuria phenotype in T1D of long duration – multivariable Random Forest analysis. All well-detectable metabolites (n = 352) were considered in the analysis. Contribution of each metabolite is shown as the mean decrease in the Gini index of the model if the metabolite were removed. Blue font marks Short and Medium Chain Fatty Acids.

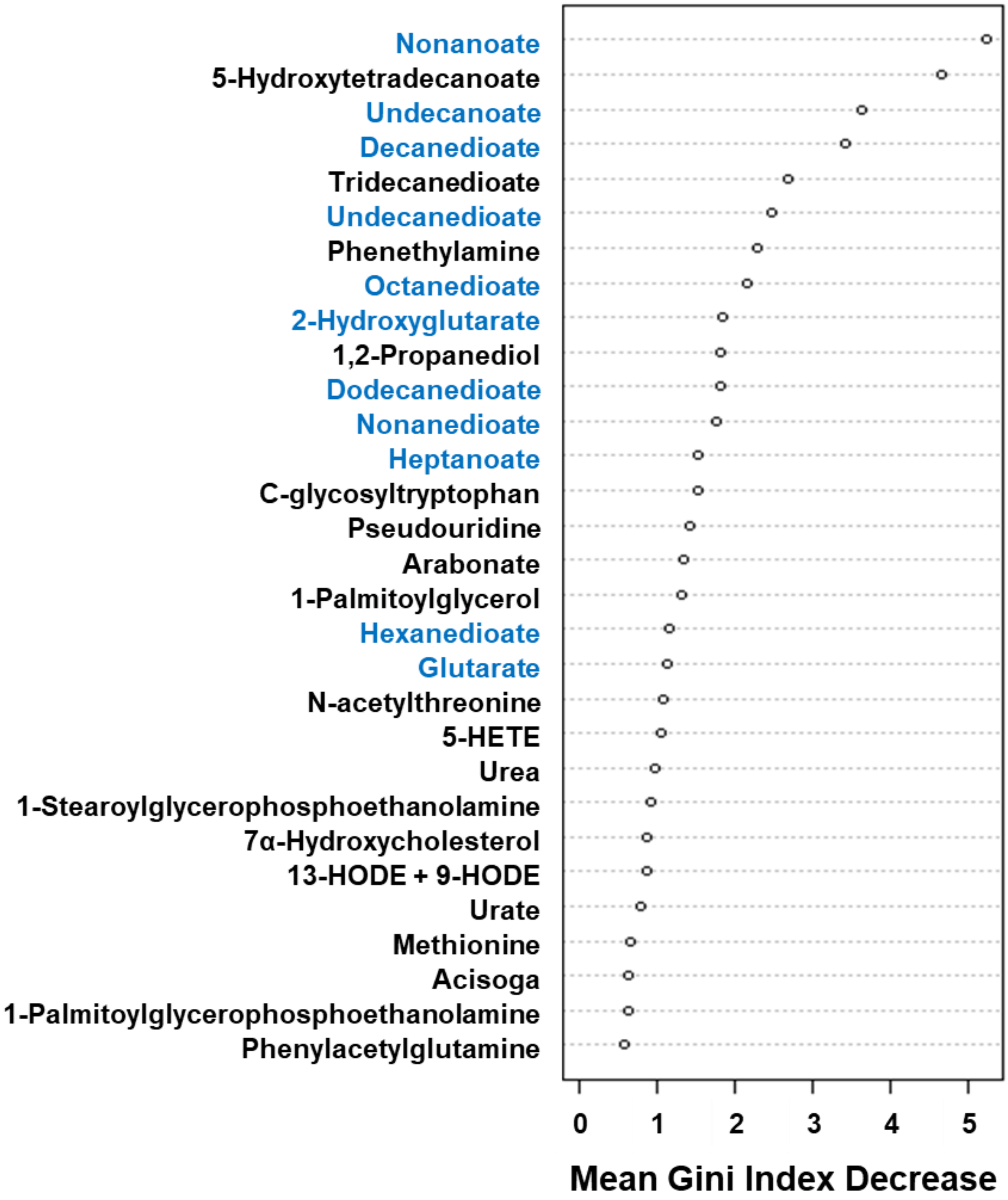

Supplementary Table S1. Fatty Acid metabolites and recent, persistent normoalbuminuria phenotype. Logistic regression analyses are presented in the crude and adjusted model by age, gender, HbA_{1c}, and eGFR. Effect sizes of well-detectable metabolites are shown per one quartile change. Effect sizes of not well-detectable metabolites are shown per the detectability threshold (please refer to the Methods Section for more detail) and marked with the italic font. Fatty Acids are ordered by chain length categories and subsequently by the number of carbons. Two asterisks mark Fatty Acids that were well detectable and highly significant in the adjusted model (Bonferroni corrected α = 0.000028). A single asterisk marks Fatty Acids that were nominally significant in the adjusted model ($p < 0.05$). Abbreviations: OR – odds ratio, CI – confidence intervals.

				Crude Model		Adjusted Model		
**Strength of Association**	**Metabolite**	**Chemical Formula**	**Double Bonds (n)**	**OR (95% CI)**	**Nominal p**	**OR (95% CI)**	**Nominal p**	**Bonferroni p**
**SHORT CHAIN FATTY ACID (SCFA)**								
**	glutarate	$C_5H_8O_4$	0	0.28 (0.21, 0.38)	2.9E-16	0.28 (0.20, 0.40)	8.4E-13	3.0E-10
**	2-hydroxyglutarate	$C_5H_8O_5$	0	0.23 (0.16, 0.32)	1.7E-17	0.25 (0.18, 0.36)	6.5E-14	2.3E-11
*	*monomethyl glutarate*	*$C_6H_{10}O_4$*	*0*	*0.08 (0.05, 0.15)*	*6.8E-18*	*0.09 (0.05, 0.17)*	*2.4E-13*	
**	4-hydroxycarboxylate	$C_4H_8O_3$	0	0.45 (0.35, 0.57)	2.1E-10	0.45 (0.34, 0.60)	3.1E-8	1.1E-5
**MEDIUM CHAIN FATTY ACID (MCFA)**								
**Dicarboxylate, odd-numbered**								
*	*heptanedioate*	*$C_7H_{12}O_4$*	*0*	*0.08 (0.04, 0.15)*	*8.8E-15*	*0.07 (0.03, 0.15)*	*2.7E-12*	
**	nonanedioate	$C_9H_{16}O_4$	0	0.20 (0.13, 0.28)	2.1E-18	0.21 (0.14, 0.31)	1.3E-14	4.5E-12
**	undecanedioate	$C_{11}H_{20}O_4$	0	0.19 (0.13, 0.28)	1.5E-18	0.19 (0.13, 0.30)	1.9E-14	6.5E-12
**Dicarboxylate, even-numbered**								
**	hexanedioate	$C_6H_{10}O_4$	0	0.33 (0.25, 0.44)	6.4E-15	0.33 (0.24, 0.46)	8.8E-12	3.1E-9
**	octanedioate	$C_8H_{14}O_4$	0	0.19 (0.13, 0.27)	1.3E-18	0.18 (0.12, 0.28)	4.1E-15	1.5E-12
**	decanedioate	$C_{10}H_{18}O_4$	0	0.16 (0.11, 0.24)	5.9E-19	0.16 (0.10, 0.25)	4.2E-15	1.5E-12
**	dodecanedioate	$C_{12}H_{22}O_4$	0	0.23 (0.17, 0.33)	1.3E-17	0.21 (0.14, 0.32)	1.8E-14	6.2E-12
**Monocarboxylate, odd-numbered**								
**	heptanoate	$C_7H_{14}O_2$	0	0.33 (0.25, 0.44)	8.5E-14	0.32 (0.23, 0.44)	2.1E-11	7.3E-9
**	nonanoate	$C_9H_{18}O_2$	0	0.13 (0.08, 0.21)	4.1E-19	0.14 (0.09, 0.23)	7.0E-16	2.5E-13
**	undecanoate	$C_{11}H_{22}O_2$	0	0.18 (0.12, 0.26)	8.5E-19	0.20 (0.13, 0.30)	5.0E-15	1.7E-12

*	10-undecenoate	$C_{11}H_{20}O_2$	1	0.74 (0.60, 0.91)	4.2E-3	0.72 (0.56, 0.91)	1.0E-2	1.0E+0
**Monocarboxylate, even-numbered**								
*	hexanoate	$C_6H_{12}O_2$	0	0.72 (0.58, 0.90)	6.1E-3	0.75 (0.59, 0.97)	4.4E-2	1.0E+0
*	*5-hydroxyhexanoate*	$C_6H_{12}O_3$	*0*	*0.18 (0.11, 0.31)*	*6.7E-10*	*0.17 (0.09, 0.32)*	*4.7E-8*	
**	octanoate	$C_8H_{16}O_2$	0	0.48 (0.37, 0.61)	2.5E-9	0.48 (0.37, 0.64)	6.3E-7	2.2E-4
	2-hydroxyoctanoate	$C_8H_{16}O_3$	0	1.06 (0.86, 1.30)	6.5E-1	0.81 (0.63, 1.04)	9.9E-2	1.0E+0
	3-hydroxyoctanoate	$C_8H_{16}O_3$	0	0.99 (0.80, 1.22)	5.8E-1	0.89 (0.70, 1.13)	2.6E-1	1.0E+0
*	*8-hydroxyoctanoate*	$C_8H_{16}O_3$	*0*	*0.03 (0.01, 0.06)*	*5.5E-16*	*0.01 (0.00, 0.04)*	*7.9E-14*	
	2-aminooctanoate	$C_8H_{17}NO_2$	0	1.13 (0.92, 1.39)	2.1E-1	0.87 (0.68, 1.12)	3.2E-1	1.0E+0
*	decanoate	$C_{10}H_{20}O_2$	0	0.60 (0.48, 0.76)	9.2E-6	0.58 (0.44, 0.76)	9.8E-5	3.5E-2
*	2-hydroxydecanoate	$C_{10}H_{20}O_3$	0	0.73 (0.59, 0.91)	3.6E-3	0.62 (0.48, 0.80)	3.1E-4	1.1E-1
*	3-hydroxydecanoate	$C_{10}H_{20}O_3$	0	0.82 (0.66, 1.01)	3.7E-2	0.78 (0.61, 0.99)	3.4E-2	1.0E+0
*	*5-hydroxydecanoate*	$C_{10}H_{20}O_3$	*0*	*0.02 (0.01, 0.05)*	*1.5E-21*	*0.01 (0.01, 0.04)*	*6.8E-17*	
*	dodecanoate	$C_{12}H_{24}O_2$	0	0.74 (0.60, 0.92)	7.8E-3	0.73 (0.56, 0.94)	2.3E-2	1.0E+0
	5-dodecenoate	$C_{12}H_{22}O_2$	1	0.85 (0.69, 1.04)	1.2E-1	0.90 (0.71, 1.15)	4.8E-1	1.0E+0
*	*5-hydroxydodecanoate*	$C_{12}H_{24}O_3$	*0*	*0.02 (0.01, 0.04)*	*8.9E-21*	*0.01 (0.00, 0.03)*	*4.6E-16*	
**LONG CHAIN FATTY ACID (LCFA)**								
**Dicarboxylate, saturated**								
**	tridecanedioate	$C_{13}H_{24}O_4$	0	0.23 (0.16, 0.32)	1.0E-17	0.22 (0.15, 0.32)	1.7E-14	5.9E-12
**	tetradecanedioate	$C_{14}H_{26}O_4$	0	0.58 (0.46, 0.73)	2.2E-6	0.49 (0.37, 0.65)	4.8E-7	1.7E-4
*	hexadecanedioate	$C_{16}H_{30}O_4$	0	0.74 (0.60, 0.92)	3.6E-3	0.63 (0.49, 0.82)	4.1E-4	1.4E-1
	octadecanedioate	$C_{18}H_{34}O_4$	0	1.17 (0.95, 1.44)	1.6E-1	1.02 (0.80, 1.30)	8.0E-1	1.0E+0
**Monocarboxylate, saturated**								
**	5-hydroxytetradecanoate	$C_{14}H_{28}O_3$	0	0.13 (0.08, 0.20)	4.3E-19	0.14 (0.08, 0.22)	1.7E-15	5.9E-13
	myristate	$C_{14}H_{28}O_2$	0	0.83 (0.67, 1.02)	8.6E-2	0.81 (0.63, 1.04)	1.4E-1	1.0E+0
	13-methylmyristic acid	$C_{15}H_{30}O_2$	*0*	*1.03 (0.64, 1.66)*	*9.0E-1*	*1.03 (0.59, 1.77)*	*9.3E-1*	
	pentadecanoate	$C_{15}H_{30}O_2$	0	1.04 (0.85, 1.28)	6.4E-1	1.02 (0.80, 1.30)	8.5E-1	1.0E+0
	palmitate	$C_{16}H_{32}O_2$	0	0.93 (0.75, 1.14)	4.9E-1	0.90 (0.71, 1.15)	4.6E-1	1.0E+0
	2-hydroxypalmitate	$C_{16}H_{32}O_3$	0	1.11 (0.90, 1.37)	3.3E-1	1.24 (0.96, 1.58)	6.8E-2	1.0E+0
	palmitate, methyl ester	$C_{17}H_{34}O_2$	0	1.03 (0.84, 1.27)	6.9E-1	1.03 (0.81, 1.32)	7.9E-1	1.0E+0
	margarate	$C_{17}H_{34}O_2$	0	0.98 (0.80, 1.20)	7.8E-1	0.91 (0.71, 1.16)	4.7E-1	1.0E+0
	stearate	$C_{18}H_{36}O_2$	0	0.94 (0.76, 1.16)	5.2E-1	0.88 (0.69, 1.13)	3.8E-1	1.0E+0
	2-hydroxystearate	$C_{18}H_{36}O_3$	0	1.08 (0.88, 1.33)	4.8E-1	1.17 (0.92, 1.50)	1.7E-1	1.0E+0

	stearamide	$C_{18}H_{37}NO$	0	1.14 (0.93, 1.41)	2.2E-1	1.05 (0.82, 1.33)	6.5E-1	1.0E+0
	stearate, methyl ester	$C_{19}H_{38}O_2$	0	0.95 (0.77, 1.16)	7.3E-1	0.98 (0.77, 1.24)	8.9E-1	1.0E+0
	nonadecanoate	$C_{19}H_{38}O_2$	0	0.94 (0.76, 1.16)	5.4E-1	0.85 (0.66, 1.09)	2.5E-1	1.0E+0
	arachidate	$C_{20}H_{40}O_2$	0	1.06 (0.86, 1.31)	5.7E-1	0.95 (0.75, 1.21)	7.9E-1	1.0E+0
**Monocarboxylate, monounsaturated**								
	myristoleate	$C_{14}H_{26}O_2$	1	0.95 (0.77, 1.17)	6.2E-1	0.99 (0.78, 1.27)	9.7E-1	1.0E+0
	palmitoleate	$C_{16}H_{30}O_2$	1	1.03 (0.84, 1.26)	7.7E-1	1.06 (0.83, 1.34)	5.9E-1	1.0E+0
	10-heptadecenoate	$C_{17}H_{32}O_2$	1	1.06 (0.86, 1.31)	5.8E-1	1.06 (0.83, 1.35)	6.3E-1	1.0E+0
	oleate	$C_{18}H_{34}O_2$	1	0.95 (0.77, 1.17)	5.8E-1	0.91 (0.71, 1.16)	4.6E-1	1.0E+0
	12,13-DiHOME	$C_{18}H_{34}O_4$	1	1.16 (0.94, 1.43)	2.2E-1	1.09 (0.86, 1.38)	5.5E-1	1.0E+0
	10-nonadecenoate	$C_{19}H_{36}O_2$	1	0.98 (0.80, 1.21)	7.7E-1	0.95 (0.75, 1.21)	6.6E-1	1.0E+0
	eicosenoate	$C_{20}H_{38}O_2$	1	0.93 (0.75, 1.14)	4.2E-1	0.90 (0.71, 1.15)	4.0E-1	1.0E+0
**Monocarboxylate, polyunsaturated**								
	linoleate	$C_{18}H_{32}O_2$	2	0.89 (0.72, 1.09)	2.1E-1	0.85 (0.66, 1.08)	1.9E-1	1.0E+0
*	linolenate (alpha or gamma)	$C_{18}H_{30}O_2$	3	0.77 (0.62, 0.95)	1.2E-2	0.74 (0.57, 0.95)	1.6E-2	1.0E+0
*	6,9,12,15-octadecatetraenoate	$C_{18}H_{28}O_2$	4	0.55 (0.44, 0.69)	3.0E-7	0.57 (0.44, 0.74)	3.1E-5	1.1E-2
*	methyl 9,12-octadecadienoate	$C_{19}H_{34}O_2$	2	0.68 (0.55, 0.85)	3.3E-4	0.68 (0.53, 0.87)	1.4E-3	5.0E-1
	dihomo-linoleate	$C_{20}H_{36}O_2$	2	0.85 (0.69, 1.04)	1.0E-1	0.82 (0.64, 1.05)	1.1E-1	1.0E+0
	dihomo-linolenate	$C_{20}H_{34}O_2$	3	1.09 (0.88, 1.34)	2.6E-1	1.15 (0.91, 1.46)	1.9E-1	1.0E+0
	arachidonate	$C_{20}H_{32}O_2$	4	1.19 (0.97, 1.47)	7.3E-2	1.22 (0.96, 1.56)	8.7E-2	1.0E+0
	eicosapentaenoate	$C_{20}H_{30}O_2$	5	0.98 (0.80, 1.21)	8.2E-1	0.99 (0.78, 1.27)	8.2E-1	1.0E+0
**VERY LONG CHAIN FATTY ACID (VLCFA)**								
	behenate	$C_{22}H_{44}O_2$	0	1.14 (0.93, 1.41)	2.5E-1	1.10 (0.86, 1.41)	4.2E-1	1.0E+0
	adrenate	$C_{22}H_{36}O_2$	4	0.94 (0.77, 1.16)	5.9E-1	0.92 (0.73, 1.17)	4.1E-1	1.0E+0
	docosadienoate	$C_{22}H_{40}O_2$	2	0.89 (0.72, 1.09)	2.9E-1	0.86 (0.68, 1.10)	2.9E-1	1.0E+0
*	docosapentaenoate (22:5n3)	$C_{22}H_{34}O_2$	5	0.84 (0.68, 1.03)	1.3E-1	0.75 (0.59, 0.97)	3.6E-2	1.0E+0
	docosapentaenoate (22:5n6)	$C_{22}H_{34}O_2$	*5*	*1.77 (1.09, 2.88)*	*2.1E-2*	*2.33 (1.31, 4.14)*	*3.8E-3*	
	docosahexaenoate	$C_{22}H_{32}O_2$	6	0.93 (0.75, 1.14)	5.9E-1	1.05 (0.82, 1.34)	5.0E-1	1.0E+0
	lignocerate	$C_{24}H_{48}O_2$	0	1.03 (0.84, 1.27)	9.9E-1	0.97 (0.76, 1.24)	6.9E-1	1.0E+0

Supplementary Figure S2. Two-way cluster analysis and heat map of protective metabolites and study subjects. Hierarchical cluster analysis – Ward method. Distances are shown. Horizontal dendrogram reflects subject-level clustering, whereas vertical dendrogram reflects metabolite-level clustering. Green horizontal dendrogram branches mark subjects protected from albuminuria. Black branches mark subjects with albuminuria. Short and Medium Chain Fatty Acids names are marked with asterisks (*) on the vertical dendrogram. In the heat map, darker red ( ) fields mark values with higher levels of the respective metabolite, and darker blue ( ) fields mark values with lower levels.

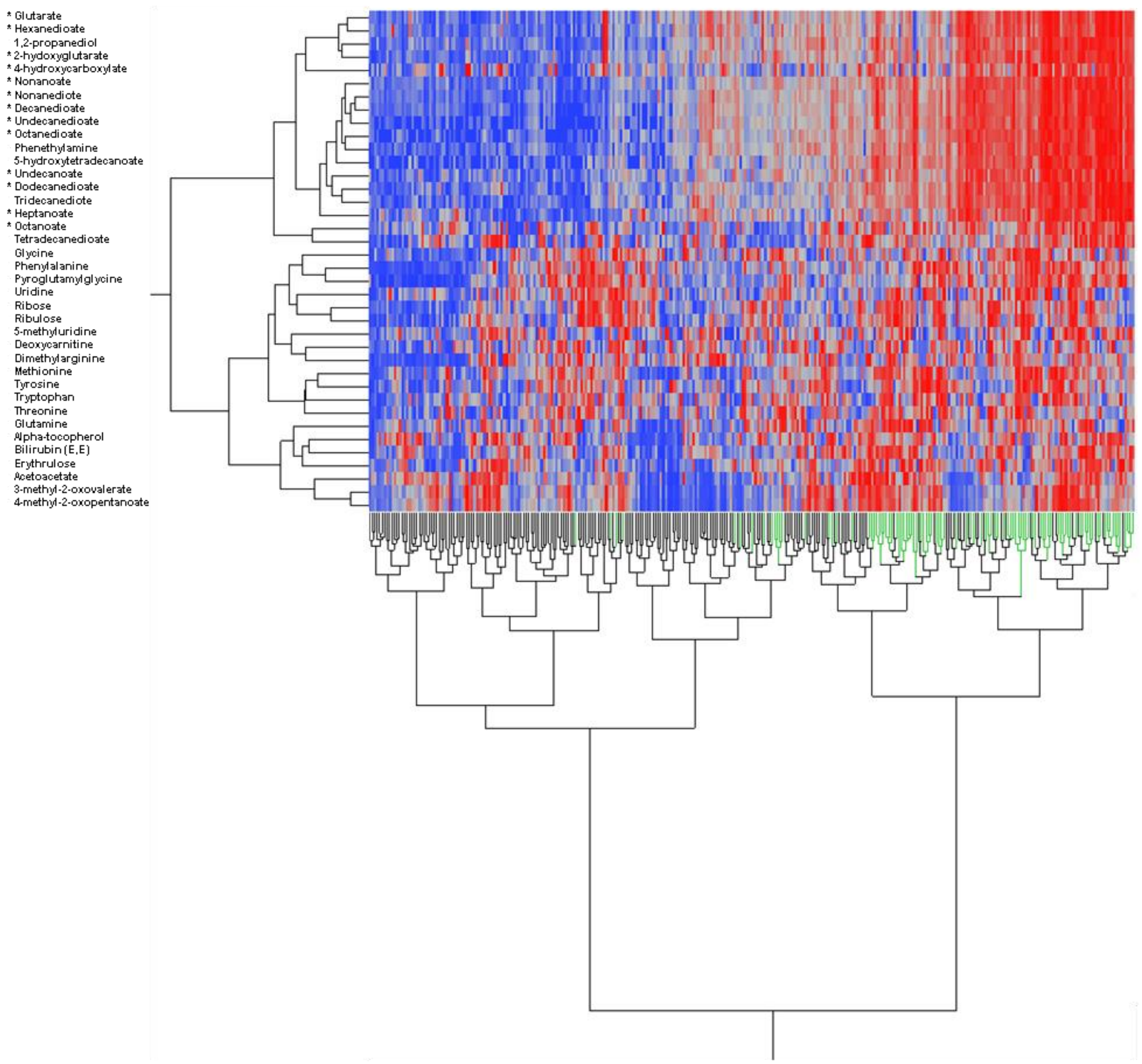

Supplementary Figure S3. Correlations of Fatty Acids with clinical measurements and biomarkers of diabetic kidney injury. Needle plot depicts the magnitude of correlations between Fatty Acids and **a**) ACR, **b**) urinary IgG2, **c**) eGFR, **d**) HbA_{1c}, **e**) plasma KIM-1, and **f**) urinary KIM-1. Spearman correlation coefficients (r) are presented on reverse y-axes for all panels but **c** (eGFR). Each needle represents an individual Fatty Acid. The order and colors of the needles follow the annotations to Figure 3. Abbreviations: ACR – albumin to creatinine ratio; HbA_{1c} – hemoglobin A_{1c}; eGFR – estimated glomerular filtration rate; IgG2 – Immunoglobulin G2; KIM-1 – Kidney Injury Molecule 1.

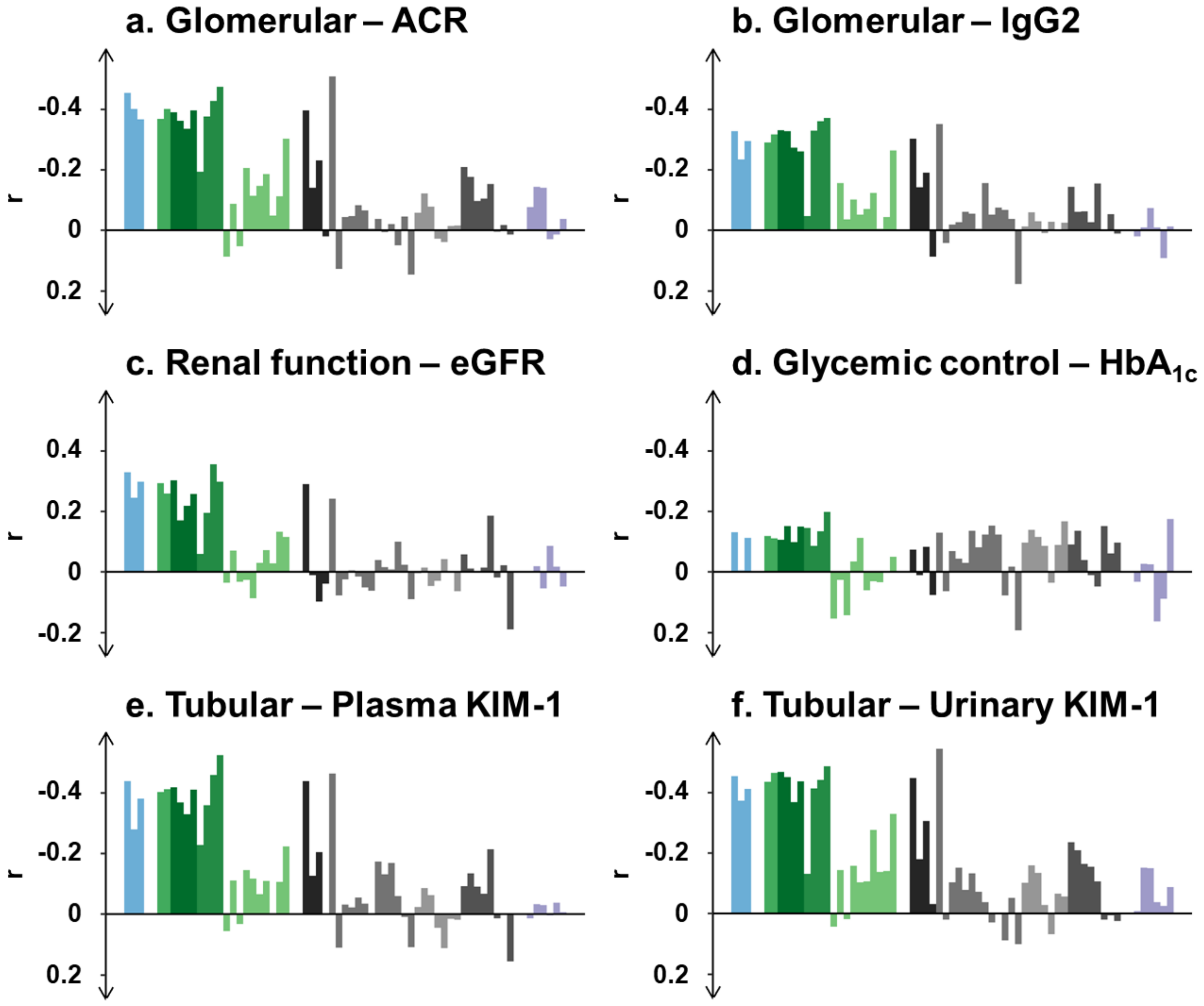

Supplementary Table S2. Metabolites robustly associated with albuminuria phenotype. Logistic regression analyses are in the crude and adjusted model by age, gender, HbA_{1c}, and eGFR. Effect sizes (odds ratios) are shown per one quartile change of the metabolite. Protective factors are presented first, followed by risk factors, and subsequently organized by biochemical class and alphabetical order. Abbreviations: HMDB – Human Metabolome Database. OR – odds ratio, CI – confidence intervals.

		Crude Model		Adjusted Model		
**Metabolite**	**HMDB ID**	**OR (95% CI)**	**Nominal p**	**OR (95% CI)**	**Nominal p**	**Bonferroni p**
**PROTECTIVE FACTORS**						
**Amino acid**						
3-methyl-2-oxovalerate	HMDB03736	0.62 (0.50, 0.78)	3.0E-05	0.50 (0.38, 0.66)	4.0E-07	1.4E-04
4-methyl-2-oxopentanoate	HMDB00695	0.54 (0.42, 0.68)	1.3E-07	0.41 (0.30, 0.55)	3.1E-09	1.1E-06
dimethylarginine	HMDB01539	0.72 (0.58, 0.89)	2.6E-03	0.55 (0.42, 0.72)	1.8E-05	6.5E-03
glutamine	HMDB00641	0.46 (0.36, 0.58)	4.0E-10	0.45 (0.34, 0.60)	2.2E-08	7.7E-06
glycine	HMDB00123	0.56 (0.44, 0.70)	4.9E-07	0.56 (0.43, 0.72)	1.2E-05	4.1E-03
methionine	HMDB00696	0.42 (0.33, 0.54)	2.4E-11	0.37 (0.27, 0.50)	1.1E-10	3.8E-08
phenethylamine	HMDB0012275	0.18 (0.12, 0.26)	2.5E-18	0.17 (0.11, 0.27)	2.7E-15	9.3E-13
phenylalanine	HMDB00159	0.55 (0.44, 0.69)	3.4E-07	0.51 (0.39, 0.67)	1.6E-06	5.5E-04
pyroglutamylglycine	HMDB0061890	0.52 (0.41, 0.66)	4.8E-08	0.52 (0.40, 0.69)	4.3E-06	1.5E-03
threonine	HMDB00167	0.56 (0.44, 0.70)	6.3E-07	0.56 (0.43, 0.73)	9.4E-06	3.3E-03
tryptophan	HMDB00929	0.62 (0.49, 0.77)	2.0E-05	0.56 (0.42, 0.73)	1.5E-05	5.4E-03
tyrosine	HMDB00158	0.58 (0.47, 0.73)	2.9E-06	0.56 (0.43, 0.73)	8.9E-06	3.1E-03
**Carbohydrate**						
erythrulose	HMDB06293	0.48 (0.38, 0.62)	3.3E-09	0.45 (0.34, 0.60)	7.1E-08	2.5E-05
ribose	HMDB00283	0.50 (0.39, 0.63)	1.1E-08	0.47 (0.36, 0.63)	1.7E-07	5.9E-05
ribulose	HMDB00621	0.54 (0.43, 0.68)	2.2E-07	0.50 (0.38, 0.65)	4.3E-07	1.5E-04
**Lipid**						
1,2-propanediol	HMDB01881	0.26 (0.19, 0.36)	1.3E-16	0.27 (0.19, 0.38)	1.8E-13	6.3E-11
2-hydroxyglutarate	HMDB00606	0.23 (0.16, 0.32)	2.5E-17	0.25 (0.18, 0.36)	6.5E-14	2.3E-11
4-hydroxycarboxylate	HMDB00710	0.45 (0.35, 0.57)	2.3E-10	0.45 (0.34, 0.60)	3.1E-08	1.1E-05
5-hydroxytetradecanoate		0.13 (0.08, 0.20)	1.3E-18	0.14 (0.08, 0.22)	1.7E-15	6.0E-13

acetoacetate	HMDB00060	0.71 (0.57, 0.88)	1.8E-03	0.55 (0.41, 0.72)	1.8E-05	6.3E-03
decanedioate	HMDB00792	0.16 (0.11, 0.24)	1.5E-18	0.16 (0.10, 0.25)	4.2E-15	1.5E-12
deoxycarnitine	HMDB01161	0.81 (0.66, 1.00)	4.9E-02	0.49 (0.36, 0.67)	2.6E-06	9.1E-04
dodecanedioate	HMDB00623	0.23 (0.17, 0.33)	3.0E-17	0.21 (0.14, 0.32)	1.8E-14	6.2E-12
glutarate	HMDB00661	0.28 (0.21, 0.38)	5.4E-16	0.28 (0.20, 0.40)	8.4E-13	3.0E-10
heptanoate	HMDB00666	0.33 (0.25, 0.44)	2.7E-14	0.32 (0.23, 0.44)	2.1E-11	7.3E-09
hexanedioate	HMDB00448	0.33 (0.25, 0.44)	1.9E-14	0.33 (0.24, 0.46)	8.8E-12	3.1E-09
nonanedioate	HMDB00784	0.20 (0.13, 0.28)	4.7E-18	0.21 (0.14, 0.31)	1.3E-14	4.5E-12
nonanoate	HMDB00847	0.13 (0.08, 0.21)	1.2E-18	0.14 (0.09, 0.23)	7.0E-16	2.5E-13
octanedioate	HMDB00893	0.19 (0.13, 0.27)	3.5E-18	0.18 (0.12, 0.28)	4.1E-15	1.5E-12
octanoate	HMDB00482	0.48 (0.37, 0.61)	2.1E-09	0.48 (0.37, 0.64)	6.3E-07	2.2E-04
tetradecanedioate	HMDB00872	0.58 (0.46, 0.73)	2.6E-06	0.49 (0.37, 0.65)	4.8E-07	1.7E-04
tridecanedioate	HMDB02327	0.23 (0.16, 0.32)	2.0E-17	0.22 (0.15, 0.32)	1.7E-14	5.9E-12
undecanedioate	HMDB00888	0.19 (0.13, 0.28)	3.6E-18	0.19 (0.13, 0.30)	1.9E-14	6.5E-12
undecanoate	HMDB00947	0.18 (0.12, 0.26)	2.4E-18	0.20 (0.13, 0.30)	5.0E-15	1.7E-12
**Nucleotide**						
5-methyluridine	HMDB00884	0.69 (0.56, 0.85)	7.2E-04	0.52 (0.40, 0.69)	5.8E-06	2.1E-03
uridine	HMDB00296	0.57 (0.46, 0.72)	1.6E-06	0.56 (0.43, 0.73)	1.3E-05	4.7E-03
**Other**						
alpha-tocopherol	HMDB01893	0.64 (0.52, 0.80)	8.8E-05	0.55 (0.42, 0.73)	1.8E-05	6.4E-03
bilirubin (E,E)	HMDB0240584	0.44 (0.35, 0.57)	1.6E-10	0.38 (0.28, 0.51)	1.9E-09	6.8E-07
**RISK FACTORS**						
**Amino acid**						
acisoga	HMDB0061384	2.01 (1.58, 2.55)	9.8E-09	1.92 (1.46, 2.52)	4.8E-06	1.7E-03
C-glycosyltryptophan	HMDB0240296	2.66 (2.03, 3.47)	8.2E-13	2.22 (1.64, 3.02)	7.6E-07	2.7E-04
gamma-glutamylisoleucine	HMDB11170	2.14 (1.67, 2.73)	1.1E-09	2.15 (1.61, 2.85)	4.2E-07	1.5E-04
gamma-glutamylleucine	HMDB11171	2.37 (1.84, 3.05)	3.1E-11	2.39 (1.77, 3.22)	3.7E-08	1.3E-05
gamma-glutamylvaline	HMDB11172	2.43 (1.88, 3.15)	1.2E-11	2.54 (1.87, 3.44)	4.4E-09	1.5E-06
leucylalanine	HMDB0028922	1.75 (1.40, 2.20)	1.3E-06	1.91 (1.45, 2.52)	1.4E-05	4.9E-03

leucylglycine	HMDB0028929	1.79 (1.42, 2.25)	6.4E-07	1.87 (1.43, 2.45)	1.3E-05	4.6E-03
leucylleucine	HMDB0028933	2.08 (1.64, 2.65)	2.6E-09	2.11 (1.60, 2.80)	3.4E-07	1.2E-04
leucylphenylalanine	HMDB0013243	2.10 (1.64, 2.67)	2.3E-09	2.43 (1.79, 3.30)	2.3E-08	8.1E-06
N-acetylthreonine	HMDB0062557	2.91 (2.20, 3.84)	6.2E-14	2.31 (1.65, 3.22)	1.9E-06	6.7E-04
phenylacetylglutamine	HMDB06344	2.12 (1.66, 2.71)	1.3E-09	1.92 (1.44, 2.54)	6.3E-06	2.2E-03
**Lipid**						
13-HODE + 9-HODE	HMDB0004667, HMDB0004670	2.99 (2.26, 3.97)	3.0E-14	2.93 (2.13, 4.03)	3.1E-11	1.1E-08
1-palmitoleoylglycerophosphocholine	HMDB0010383	1.73 (1.38, 2.17)	2.0E-06	1.98 (1.50, 2.61)	2.4E-06	8.6E-04
1-palmitoylglycerol	HMDB0011564	1.97 (1.55, 2.49)	2.0E-08	2.10 (1.59, 2.78)	2.9E-07	1.0E-04
1-palmitoylglycerophosphoethanolamine	HMDB11503	2.07 (1.63, 2.64)	3.1E-09	2.68 (1.96, 3.67)	3.6E-10	1.3E-07
1-palmitoylglycerophosphoinositol	HMDB0061695	1.55 (1.25, 1.93)	8.6E-05	1.79 (1.38, 2.33)	7.9E-06	2.8E-03
1-stearoylglycerophosphoethanolamine	HMDB11130	2.08 (1.64, 2.65)	2.5E-09	2.09 (1.58, 2.77)	5.1E-07	1.8E-04
1-stearoylglycerophosphoinositol	HMDB0240261	1.85 (1.47, 2.33)	1.8E-07	2.18 (1.65, 2.90)	4.0E-08	1.4E-05
2-palmitoylglycerophosphoethanolamine	HMDB0011473	1.83 (1.45, 2.30)	2.9E-07	2.19 (1.65, 2.92)	2.5E-08	8.8E-06
3-methylbutyrate	HMDB00718	1.86 (1.48, 2.35)	1.5E-07	1.94 (1.48, 2.55)	1.9E-06	6.5E-04
5-HETE	HMDB11134	3.35 (2.49, 4.52)	1.8E-15	3.79 (2.65, 5.42)	2.9E-13	1.0E-10
7-alpha-hydroxycholesterol	HMDB01496	2.88 (2.18, 3.80)	8.2E-14	3.02 (2.20, 4.15)	5.2E-12	1.8E-09
7-beta-hydroxycholesterol	HMDB06119	2.56 (1.97, 3.33)	2.4E-12	2.79 (2.05, 3.79)	5.0E-11	1.7E-08
glycerophosphorylcholine	HMDB00086	1.93 (1.52, 2.44)	4.2E-08	1.98 (1.50, 2.61)	1.1E-06	4.0E-04
**Nucleotide**						
pseudouridine	HMDB00767	2.94 (2.22, 3.89)	4.7E-14	2.58 (1.84, 3.60)	9.7E-08	3.4E-05
**Other**						
arabonate	HMDB00539	2.77 (2.11, 3.64)	2.3E-13	2.12 (1.57, 2.87)	6.9E-07	2.4E-04
phosphate	HMDB01429	1.75 (1.40, 2.20)	1.3E-06	1.83 (1.38, 2.41)	2.0E-05	7.1E-03

Supplementary Table S3. Patterns of metabolites associations from the FinnDiane Study [23] and our current study of Joslin Kidney Study participants. Forty two metabolites reported in both studies are ordered by biochemical groups. The pattern of a metabolite indicates whether it was associated with "risk" or "protection" for progression to microalbuminuria (FinnDiane) or albuminuria phenotype (Joslin). This analysis of the Joslin data relied on the alpha = 0.05. Effect estimates of the cross-sectional comparison in the Joslin Kidney Study are shown per quartile change.

		FinnDiane Study	Joslin Kidney Study		
		Baseline normoalbuminuria	Normoalbuminuria vs. albuminuria		
		Longitudinal	Cross-sectional		
**Metabolite**	**HMDB ID**	**Pattern**	**Pattern**	**OR (95% CI)**	**p**
**UREMIC SOLUTE**					
C-glycosyltryptophan	HMDB0240296	Risk	Risk	2.66 (2.03, 3.47)	8.2E-13
N-acetylalanine	HMDB00766	Risk	Risk	1.49 (1.20, 1.85)	3.3E-04
mannitol	HMDB00765	Risk	Risk	1.24 (1.00, 1.53)	4.7E-02
sorbitol	HMDB00247	Risk		0.97 (0.78, 1.19)	7.4E-01
xylonate	HMDB0060256	Risk	Risk	1.47 (1.18, 1.82)	5.4E-04
N2,N2-dimethylguanosine	HMDB04824	Risk		0.99 (0.80, 1.22)	9.2E-01
N6-carbamoylthreonyladenosine	HMDB0041623	Risk	Risk	1.87 (1.48, 2.36)	1.7E-07
pseudouridine	HMDB00767	Risk	Risk	2.94 (2.22, 3.89)	4.7E-14
urate	HMDB00289	Risk	Risk	2.21 (1.73, 2.84)	3.0E-10
**LIPID**					
**Carnitine**					
carnitine	HMDB0000062	Risk	Risk	1.34 (1.08, 1.66)	6.8E-03
octanoylcarnitine	HMDB00791	Risk		1.13 (0.92, 1.39)	2.4E-01
**Fatty acid (straight chain)**					
glutarate	HMDB00661	Risk	Protect	0.28 (0.21, 0.38)	5.4E-16
octanedioate	HMDB00893	Risk	Protect	0.19 (0.13, 0.27)	3.5E-18
pentadecanoate	HMDB00826	Risk		1.04 (0.85, 1.28)	6.9E-01
docosadienoate	HMDB0061714	Risk		0.89 (0.72, 1.09)	2.6E-01

**Fatty acid (branched chain)**					
2-aminoheptanoic acid	HMDB0094649	Risk	Risk	1.28 (1.04, 1.58)	2.2E-02
hydroxybutyrylcarnitine	HMDB13127	Risk	Risk	1.53 (1.23, 1.91)	1.5E-04
**Glycerol**					
1,3-dipalmitoylglycerol	HMDB0056009	Risk	Risk	1.55 (1.24, 1.93)	9.8E-05
1-stearoylglycerol	HMDB0011131	Risk	Risk	1.72 (1.37, 2.16)	2.4E-06
glycerol	HMDB00131	Risk		0.86 (0.70, 1.06)	1.6E-01
**Lysolipid**					
1-palmitoylglycerophosphoethanolamine	HMDB11503	Risk	Risk	2.07 (1.63, 2.64)	3.1E-09
1-stearoylglycerophosphoethanolamine	HMDB11130	Risk	Risk	2.08 (1.64, 2.65)	2.5E-09
2-palmitoylglycerophosphoethanolamine	HMDB0011473	Risk	Risk	1.83 (1.45, 2.30)	2.9E-07
1-docosahexaenoylglycerophosphocholine	HMDB0010404	Protect		1.01 (0.82, 1.24)	9.2E-01
**Oxysterol**					
7-alpha-hydroxycholesterol	HMDB01496	Protect	Risk	2.88 (2.18, 3.80)	8.2E-14
7-beta-hydroxycholesterol	HMDB06119	Protect	Risk	2.56 (1.97, 3.33)	2.4E-12
**Other steroid**					
cortisone	HMDB02802	Risk		1.10 (0.90, 1.36)	3.6E-01
pregnen-diol disulfate	HMDB0094650	Risk	Risk	1.85 (1.47, 2.34)	1.8E-07
**AMINO ACID**					
**Dipeptide**					
pro-hydroxy-pro	HMDB06695	Risk		1.11 (0.90, 1.36)	3.4E-01
gamma-glutamylleucine	HMDB11171	Protect	Risk	2.37 (1.84, 3.05)	3.1E-11
**Other amino acid**					
glutamine	HMDB00641	Protect	Protect	0.46 (0.36, 0.58)	4.0E-10
indolepropionate	HMDB02302	Protect	Protect	0.75 (0.60, 0.92)	7.3E-03
**CARBOHYDRATE**					
**Monosaccharide**					
1,5-anhydroglucitol	HMDB02712	Protect	Protect	0.80 (0.65, 0.99)	4.0E-02
**OTHER**					

gamma-tocopherol	HMDB01492	Risk	1.09 (0.89, 1.35)	3.9E-01
malate	HMDB00156	Risk	1.05 (0.85, 1.29)	6.5E-01
succinate	HMDB00254	Risk	1.19 (0.97, 1.47)	1.0E-01
lactate	HMDB00190	Risk	1.00 (0.82, 1.23)	9.7E-01
2-hydroxybutyrate	HMDB00008	Risk	1.11 (0.90, 1.37)	3.2E-01
alanine	HMDB00161	Risk	0.83 (0.67, 1.02)	8.3E-02
pipecolate	HMDB00070	Risk	1.20 (0.97, 1.48)	8.6E-02
taurocholenate sulfate		Risk	0.97 (0.79, 1.19)	7.8E-01
5-oxoproline	HMDB00267	Protect	1.14 (0.93, 1.41)	2.1E-01

Supplementary Table S4. Frequency of detection in global metabolomic profiling. Detectability is presented as the percentage of subjects in which the metabolite was quantified. Metabolites are organized by biochemical class and subsequently in alphabetical order. 580 metabolites were detected in at least one subject in our study population. Drug derivatives and xenobiotics are not shown. Abbreviation: HMDB – Human Metabolome Database.

Metabolite	HMDB ID	Detectability (%)
**Amino acid**		
1-methylhistidine	HMDB00001	94
2-aminoadipate	HMDB00510	90
2-aminobutyrate	HMDB00650	100
2-hydroxy-3-methylvalerate	HMDB00317	87
2-hydroxybutyrate (AHB)	HMDB00008	100
2-methylbutyrylcarnitine (C5)	HMDB00378	100
3-(3-hydroxyphenyl)propionate	HMDB00375	74
3-(4-hydroxyphenyl)lactate	HMDB00755	100
3-hydroxy-2-ethylpropionate	HMDB00396	100
3-hydroxyisobutyrate	HMDB00336	99
3-indoxyl sulfate	HMDB00682	100
3-methoxytyrosine	HMDB01434	100
3-methyl-2-oxobutyrate	HMDB00019	94
3-methyl-2-oxovalerate	HMDB03736	100
3-methylglutarylcarnitine (C6)	HMDB00552	93
3-methylhistidine	HMDB00479	98
3-phenylpropionate (hydrocinnamate)	HMDB00764	77
4-hydroxyphenylacetate	HMDB00020	100
4-methyl-2-oxopentanoate	HMDB00695	100
5-hydroxymethyl-2-furoic acid	HMDB02432	81
5-oxoproline	HMDB00267	100
acisoga		100
ADpSGEGDFXAEGGGVR		1
ADSGEGDFXAEGGGVR		90
agmatine	HMDB01432	0.3
alanine	HMDB00161	100
alanylalanine		77
alanylleucine		65
alpha-hydroxyisocaproate	HMDB00746	98
alpha-hydroxyisovalerate	HMDB00407	100
alpha-ketobutyrate	HMDB00005	70
arginine	HMDB00517	99
asparagine	HMDB00168	100

asparagylleucine		75
aspartate	HMDB00191	100
aspartylleucine		77
aspartylphenylalanine	HMDB00706	91
beta-alanine	HMDB00056	100
beta-hydroxyisovalerate	HMDB00754	100
beta-hydroxypyruvate	HMDB01352	96
betaine	HMDB00043	100
bradykinin	HMDB04246	34
bradykinin, des-arg(9)	HMDB04246	71
bradykinin, hydroxy-pro(3)	HMDB11728	19
C-glycosyltryptophan		100
citrulline	HMDB00904	100
creatine	HMDB00064	100
creatinine	HMDB00562	100
cyclo(leu-pro)		84
cysteine	HMDB00574	92
dimethylarginine (SDMA + ADMA)	HMDB01539	100
dimethylglycine	HMDB00092	100
DSGEGDFXAEGGGVR		91
gamma-glutamylalanine		96
gamma-glutamylglutamine	HMDB11738	98
gamma-glutamylisoleucine	HMDB11170	100
gamma-glutamylleucine	HMDB11171	100
gamma-glutamylmethionine		97
gamma-glutamylphenylalanine	HMDB00594	100
gamma-glutamyltyrosine		100
gamma-glutamylvaline	HMDB11172	100
glutamate	HMDB03339	100
glutamine	HMDB00641	98
glutamine-leucine		76
glutarylcarnitine (C5)	HMDB13130	99
glycine	HMDB00123	100
glycylleucine	HMDB00759	66
glycylphenylalanine		90
glycylvaline		92
GSPAXNVAVHVFR		0.1
histidine	HMDB00177	100
histidylalanine		84
histidylleucine		75
histidylphenylalanine		54
histidylserine		52
histidyltryptophan		87

homocitrulline	HMDB00679	97
homocysteine	HMDB00742	48
HWESASLLR		43
HWESASXX		99
HXGXA		86
indoleacetate	HMDB00197	100
indoleacetylglutamine		28
indolelactate	HMDB00671	100
indolepropionate	HMDB02302	97
isobutyrylcarnitine	HMDB00736	100
isoleucine	HMDB00172	100
isoleucylthreonine		92
isovalerylcarnitine	HMDB00688	100
kynurenate	HMDB00715	90
kynurenine	HMDB00684	100
leucine	HMDB00687	100
leucylalanine		96
leucylglutamate		62
leucylglycine		92
leucylhistidine		13
leucylleucine		98
leucylphenylalanine		85
leucylserine		83
leucyltyrosine		0.1
lysine	HMDB00182	100
methionine	HMDB00696	100
N6-acetyllysine	HMDB00206	100
N-acetylalanine	HMDB00766	100
N-acetylaspartate (NAA)	HMDB00812	100
N-acetyl-beta-alanine		100
N-acetylcarnosine		100
N-acetylglycine	HMDB00532	100
N-acetylmethionine	HMDB11745	81
N-acetylserine	HMDB02931	100
N-acetylthreonine		100
N-formylmethionine	HMDB01015	98
N-methyl proline		93
o-cresol sulfate		89
ornithine	HMDB03374	100
p-cresol sulfate	HMDB11635	100
phenethylamine		90
phenol sulfate		100
phenylacetate	HMDB00209	89

phenylacetylglutamine	HMDB06344	100
phenylalanine	HMDB00159	100
phenylalanylalanine		12
phenylalanylarginine		8
phenylalanylglutamate		81
phenylalanylleucine		73
phenylalanylmethionine		25
phenylalanylphenylalanine		94
phenylalanylserine		67
phenylalanyltryptophan		90
phenyllactate (PLA)	HMDB00779	97
picolinate	HMDB02243	0.1
pipecolate	HMDB00070	100
pro-hydroxy-pro	HMDB06695	100
proline	HMDB00162	100
putrescine	HMDB01414	0.6
pyroglutamine		100
pyroglutamylglycine		92
pyroglutamylvaline		40
sarcosine (N-Methylglycine)	HMDB00271	94
serine	HMDB00187	100
serotonin (5HT)	HMDB00259	97
serylleucine		91
S-methylcysteine	HMDB02108	100
threonine	HMDB00167	99
threonylglutamate		67
threonylphenylalanine		86
tiglyl carnitine	HMDB02366	87
trans-4-hydroxyproline	HMDB00725	100
tryptophan	HMDB00929	100
tryptophan betaine		99
tryptophylasparagine		73
tryptophylglutamate		88
tyrosine	HMDB00158	100
tyrosylglutamate		58
tyrosyltryptophan		14
urea	HMDB00294	100
valine	HMDB00883	100
valylarginine		30
valylphenylalanine		42
XHWESASXXR		17

Carbohydrate

1,3-dihydroxyacetone	HMDB01882	100
1,5-anhydroglucitol (1,5-AG)	HMDB02712	99
1,6-anhydroglucose	HMDB00640	99
3-phosphoglycerate	HMDB00807	18
arabinose	HMDB00646	70
arabitol	HMDB01851	100
erythronate	HMDB00613	100
erythrulose	HMDB06293	98
fructose	HMDB00660	100
fucose	HMDB00174	98
galactitol (dulcitol)	HMDB00107	75
gluconate	HMDB00625	100
glucose	HMDB00122	100
glucose-6-phosphate (G6P)	HMDB01401	64
glycerate	HMDB00139	100
lactate	HMDB00190	100
maltose	HMDB00163	89
maltotriose	HMDB01262	67
mannitol	HMDB00765	100
mannose	HMDB00169	100
methyl-beta-glucopyranoside		22
N-acetylneuraminate	HMDB00230	100
pyruvate	HMDB00243	100
ribitol	HMDB00508	100
ribose	HMDB00283	100
ribulose	HMDB00621	96
sorbitol	HMDB00247	100
threitol	HMDB04136	100
trehalose	HMDB00975	53
xylitol	HMDB02917	100
xylonate		100
xylose	HMDB00098	100
**Lipid**		
1,2-dipalmitoylglycerol	HMDB07098	99
1,2-propanediol	HMDB01881	100
1,3-dipalmitoylglycerol		100
10-heptadecenoate		100
10-nonadecenoate		100
10-undecenoate		100
12,13-hydroxyoctadec-9(Z)-enoate	HMDB04705	85
13-HODE + 9-HODE		99
13-methylmyristic acid		62

15-methylpalmitate (isobar with 2-methylpalmitate)		92
17-methylstearate		98
1-arachidonoylglycerophosphocholine (20:4n6)		100
1-arachidonoylglycerophosphoethanolamine	HMDB11517	100
1-arachidonoylglycerophosphoinositol		100
1-arachidonoylglyercophosphate		96
1-arachidonylglycerol	HMDB11572	96
1-dihomo-linoleoylglycerophosphocholine (20:2n6)		82
1-docosahexaenoylglycerophosphocholine (22:6n3)		99
1-docosahexaenoylglycerophosphoethanolamine		100
1-docosapentaenoylglycerophosphocholine (22:5)		76
1-eicosapentaenoylglycerophosphocholine (20:5n3)		90
1-eicosatrienoylglycerophosphocholine (20:3)		100
1-eicosatrienoylglycerophosphoethanolamine		100
1-eicosenoylglycerophosphocholine (20:1n9)		74
1-linolenoylglycerol	HMDB11569	86
1-linolenoylglycerophosphocholine (18:3n3)		95
1-linoleoylglycerol (1-monolinolein)		100
1-linoleoylglycerophosphocholine (18:2n6)		100
1-linoleoylglycerophosphoethanolamine	HMDB11507	100
1-margaroylglycerophosphocholine (17:0)	HMDB12108	96
1-myristoylglycerol (1-monomyristin)	HMDB11561	95
1-myristoylglycerophosphocholine (14:0)	HMDB10379	99
1-oleoylglycerol (1-monoolein)	HMDB11567	100
1-oleoylglycerophosphocholine (18:1)		100
1-oleoylglycerophosphoethanolamine	HMDB11506	100
1-oleoylglycerophosphoinositol		96
1-oleoylplasmenylethanolamine		97
1-palmitoleoylglycerophosphocholine (16:1)		100
1-palmitoylglycerol (1-monopalmitin)		100
1-palmitoylglycerophosphate	HMDB00327	94
1-palmitoylglycerophosphocholine (16:0)		100
1-palmitoylglycerophosphoethanolamine	HMDB11503	100
1-palmitoylglycerophosphoinositol		95
1-palmitoylplasmenylethanolamine		100
1-stearoylglycerol (1-monostearin)		100
1-stearoylglycerophosphocholine (18:0)		100
1-stearoylglycerophosphoethanolamine	HMDB11130	100
1-stearoylglycerophosphoinositol		100
21-hydroxypregnenolone disulfate	HMDB04026	97
2-aminoheptanoic acid		99
2-aminooctanoate	HMDB00991	100
2-arachidonoylglycerophosphoethanolamine		94

2-docosahexaenoylglycerophosphoethanolamine		64
2-hydroxydecanoate		100
2-hydroxyglutarate	HMDB00606	100
2-hydroxyoctanoate	HMDB02264	95
2-hydroxypalmitate		100
2-hydroxystearate		100
2-linoleoylglycerol (2-monolinolein)	HMDB11538	86
2-linoleoylglycerophosphocholine		75
2-linoleoylglycerophosphoethanolamine		98
2-oleoylglycerol (2-monoolein)		97
2-oleoylglycerophosphocholine		63
2-oleoylglycerophosphoethanolamine		99
2-palmitoylglycerol (2-monopalmitin)		100
2-palmitoylglycerophosphocholine		79
2-palmitoylglycerophosphoethanolamine		100
2-stearoylglycerophosphocholine		96
3-carboxy-4-methyl-5-propyl-2-furanpropanoate (CMPF)		100
3-dehydrocarnitine	HMDB12154	100
3-hydroxybutyrate (BHBA)	HMDB00357	100
3-hydroxydecanoate	HMDB02203	95
3-hydroxyoctanoate	HMDB01954	92
3-hydroxypropanoate	HMDB00700	100
3-methylbutyrate	HMDB00718	97
4-androsten-3beta,17beta-diol disulfate 1	HMDB03818	100
4-androsten-3beta,17beta-diol disulfate 2	HMDB03818	100
4-hydroxycarboxylate	HMDB00710	99
5alpha-androstan-3alpha,17beta-diol disulfate		38
5alpha-androstan-3beta,17alpha-diol disulfate		61
5alpha-androstan-3beta,17beta-diol disulfate	HMDB00493	97
5alpha-pregnan-3alpha,20beta-diol disulfate 1		37
5alpha-pregnan-3beta,20alpha-diol disulfate		96
5-dodecenoate	HMDB00529	89
5-HEPE	HMDB05081	68
5-HETE	HMDB11134	85
5-hydroxydecanoate		47
5-hydroxydodecanoate		48
5-hydroxyhexanoate	HMDB00525	75
5-hydroxytetradecanoate		81
5-KETE	HMDB10217	50
6,9,12,15-octadecatetraenoate	HMDB06547	93
7-alpha-hydroxy-3-oxo-4-cholestenoate (7-Hoca)	HMDB12458	100
7-alpha-hydroxycholesterol	HMDB01496	81

7-beta-hydroxycholesterol	HMDB06119	100
7-ketodeoxycholate	HMDB00391	11
8-hydroxyoctanoate	HMDB00711	62
acetoacetate	HMDB00060	100
acetylcarnitine	HMDB00201	97
adrenate	HMDB02226	100
andro steroid monosulfate 2	HMDB02759	100
androsterone sulfate	HMDB02759	99
arachidate	HMDB02212	100
arachidonate	HMDB01043	100
behenate	HMDB00944	99
beta-sitosterol	HMDB00852	82
butyrylcarnitine	HDMB02013	97
campesterol	HMDB02869	91
carnitine		100
chiro-inositol		90
cholate	HMDB00619	75
cholesterol	HMDB00067	100
choline		100
cortisol	HMDB00063	100
cortisone	HMDB02802	98
decanedioate	HMDB00792	93
decanoate	HMDB00511	100
decanoylcarnitine	HMDB00651	98
dehydroisoandrosterone sulfate (DHEA-S)	HMDB01032	100
deoxycarnitine	HMDB01161	100
deoxycholate	HMDB00626	75
dihomo-linoleate		100
dihomo-linolenate	HMDB02925	100
docosadienoate		98
docosahexaenoate	HMDB02183	100
docosapentaenoate (22:5n3)	HMDB01976	100
docosapentaenoate (22:5n6)	HMDB13123	77
dodecanedioate	HMDB00623	98
dodecanoate	HMDB00638	100
eicosapentaenoate	HMDB01999	100
eicosenoate	HMDB02231	100
epiandrosterone sulfate	HMDB00365	99
estrone 3-sulfate	HMDB01425	21
glutarate	HMDB00661	100
glycerol	HMDB00131	100
glycerol 3-phosphate (G3P)	HMDB00126	100
glycerophosphorylcholine (GPC)	HMDB00086	100

glycochenodeoxycholate	HMDB00637	91
glycocholate	HMDB00138	100
glycocholenate sulfate		100
glycodeoxycholate	HMDB00631	82
glycolithocholate sulfate	HMDB02639	99
heptanedioate	HMDB00857	80
heptanoate	HMDB00666	99
hexadecanedioate	HMDB00672	100
hexanedioate	HMDB00448	100
hexanoate	HMDB00535	98
hexanoylcarnitine	HMDB00705	95
hydroxybutyrylcarnitine	HMDB13127	84
lathosterol	HMDB01170	94
laurylcarnitine	HMDB02250	56
leukotriene B4	HMDB01085	76
lignocerate	HMDB02003	96
linoleate	HMDB00673	100
linolenate (alpha or gamma)	HMDB01388	100
margarate	HMDB02259	100
methyl 9,12-octadecadienoate		87
monomethyl glutarate	HMDB00858	38
myo-inositol	HMDB00211	100
myristate	HMDB00806	100
myristoleate	HMDB02000	100
nonadecanoate	HMDB00772	100
nonanedioate	HMDB00784	99
nonanoate	HMDB00847	100
octadecanedioate	HMDB00782	97
octanedioate	HMDB00893	85
octanoate	HMDB00482	100
octanoylcarnitine	HMDB00791	99
oleate	HMDB00207	100
oleoylcarnitine	HMDB05065	97
palmitate	HMDB00220	100
palmitate, methyl ester		100
palmitoleate	HMDB03229	100
palmitoyl sphingomyelin		100
palmitoylcarnitine	HMDB00222	94
pentadecanoate	HMDB00826	100
pregn steroid monosulfate		100
pregnanediol-3-glucuronide		95
pregnen-diol disulfate		100
pregnenolone sulfate	HMDB00774	99

propionylcarnitine	HMDB00824	100
prostaglandin E2	HMDB01220	51
scyllo-inositol	HMDB06088	100
sphingosine	HMDB00252	76
stearamide		98
stearate	HMDB00827	100
stearate, methyl ester		100
stearoyl sphingomyelin	HMDB01348	100
stearoylcarnitine	HMDB00848	94
taurochenodeoxycholate	HMDB00951	62
taurocholate	HMDB00036	90
taurocholenate sulfate		98
taurodeoxycholate	HMDB00896	72
taurolithocholate 3-sulfate	HMDB02580	92
tauroursodeoxycholate	HMDB00874	37
tetradecanedioate	HMDB00872	98
tridecanedioate	HMDB02327	95
undecanedioate	HMDB00888	83
undecanoate	HMDB00947	100
**Nucleotide**		
3-aminoisobutyrate	HMDB03911	100
5,6-dihydrouracil	HMDB00076	100
5-methyluridine (ribothymidine)	HMDB00884	95
7-methylguanine	HMDB00897	98
adenine	HMDB00034	47
adenosine	HMDB00050	25
adenosine 5'-monophosphate (AMP)	HMDB00045	48
allantoin	HMDB00462	100
dihydroorotate	HMDB03349	95
guanosine	HMDB00133	47
hypoxanthine	HMDB00157	99
inosine	HMDB00195	89
N1-methyladenosine	HMDB03331	99
N2,N2-dimethylguanosine	HMDB04824	88
N6-carbamoylthreonyladenosine		85
N6-methyladenosine	HMDB04044	52
pseudouridine	HMDB00767	100
uracil	HMDB00300	100
urate	HMDB00289	100
uridine	HMDB00296	100
xanthine	HMDB00292	100

**Other**		
2,3-dihydroxyisovalerate		57
alpha-CEHC glucuronide		24
alpha-ketoglutarate	HMDB00208	94
alpha-tocopherol	HMDB01893	100
arabonate	HMDB00539	100
ascorbate (Vitamin C)	HMDB00044	0.1
beta-tocopherol	HMDB06335	60
bilirubin (E,E)		99
bilirubin (Z,Z)	HMDB00054	73
biliverdin	HMDB01008	74
citrate	HMDB00094	100
delta-tocopherol	HMDB02902	94
flavin adenine dinucleotide (FAD)	HMDB01248	51
gamma-CEHC	HMDB01931	95
gamma-CEHC glucuronide		69
gamma-tocopherol	HMDB01492	99
gulono-1,4-lactone	HMDB03466	100
heme	HMDB03178	69
L-urobilin	HMDB04159	50
malate	HMDB00156	100
N1-Methyl-2-pyridone-5-carboxamide	HMDB04193	98
nicotinamide	HMDB01406	97
nicotinurate	HMDB03269	0.3
pantothenate	HMDB00210	100
phosphate	HMDB01429	100
pyridoxate	HMDB00017	100
pyrophosphate (PPi)	HMDB00250	97
quinolinate	HMDB00232	84
succinate	HMDB00254	100
succinylcarnitine		99
threonate	HMDB00943	100
trigonelline (N'-methylnicotinate)	HMDB00875	81
urobilinogen	HMDB04157	60
